# Transperineal Laser Ablation for Treatment of Lower Urinary Tract Symptoms in Benign Prostate Enlargement: A Systematic Review and Meta-analysis

**DOI:** 10.1590/S1677-5538.IBJU.2025.0423

**Published:** 2025-05-20

**Authors:** Iago Zang Pires, Marília Oberto da Silva Gobbo, Alexandre Yamada Fujimura, Tanize Louize Milbradt, Renan Yuji Ura Sudo, Mable Pereira, Nilson Marquardt, Gustavo Franco Carvalhal, Márcio Augusto Averbeck

**Affiliations:** 1 Pontifícia Universidade Católica do Rio Grande do Sul Divisão de Medicina Porto Alegre RS Brasil Divisão de Medicina, Pontifícia Universidade Católica do Rio Grande do Sul (PUCRS), Porto Alegre, RS, Brasil; 2 Faculdade de Medicina de Marília Divisão de Medicina Marília SP Brasil Divisão de Medicina, Faculdade de Medicina de Marília (FAMEMA), Marília, SP, Brasil; 3 Universidade Federal de Santa Maria Divisão de Medicina Santa Maria RS Brasil Divisão de Medicina, Universidade Federal de Santa Maria (UFSM), Santa Maria, RS, Brasil; 4 Universidade Federal da Grande Dourados Divisão de Medicina Dourados MS Brasil Divisão de Medicina, Universidade Federal da Grande Dourados (UFGD), Dourados, MS, Brasil; 5 Lincoln American University School of Medicine Divisão de Medicina Georgetown Guiana Divisão de Medicina, Lincoln American University School of Medicine, Georgetown, Guiana; 6 Pontifícia Universidade Católica do Rio Grande do Sul Hospital São Lucas Departamento de Urologia Porto Alegre RS Brasil Departamento de Urologia, Hospital São Lucas, Pontifícia Universidade Católica do Rio Grande do Sul (PUCRS), Porto Alegre, RS, Brasil

**Keywords:** Prostatic Hyperplasia, Transurethral Resection of Prostate, Laser Therapy

## Abstract

**Purpose:**

This is a systematic review and meta-analysis of the outcomes of transperineal prostate laser ablation (TPLA) in men with benign prostatic enlargement.

**Materials and Methods:**

Pubmed, Embase, Scopus, and Cochrane Library databases were searched from inception to July 2024. Random-effects model was employed to compute mean differences for continuous endpoints. Heterogeneity was evaluated by prediction interval and I-squared statistics. Results were reported following the PRISMA guidelines.

**Results:**

Seventeen studies involving 777 patients with mean age of 62 to 80 years were included. Over 12-month follow-up, TPLA decreased the International Prostate Symptom Score (MD −12.62; 95% CI −14.87 to −10.37; p<0.001; I2 = 90%), post-void residual (MD −73.24 mL; 95% CI −96.91 to −49.57; p<0.001; I2 = 89%), and prostate volume (MD −21.23 mL; 95% CI −32.65 to −9.81; p<0.001; I2 = 84%). TPLA increased the maximum urinary flow rate (MD 6.32 mL/s; 95% CI 4.69 to 7.95; p<0.001; I2 = 81%). Ejaculatory and erectile functions were not impacted. Compared to TURP, TPLA was associated with ejaculatory function preservation, shorter operating time and length of stay. Risk of bias for the non-randomized studies was moderate, and low for the randomized studies.

**Conclusions:**

TPLA demonstrated favorable outcomes for BPE without a negative impact on sexual function. This minimally invasive treatment was found to have advantages over TURP, such as, ejaculatory function preservation, reduced operative time, and shorter hospital stay. Evidence for this MIST is emerging but remains predominantly retrospective with short follow-up, highlighting the need for further comparative prospective studies.

## INTRODUCTION

Benign Prostatic Enlargement (BPE) frequently causes lower urinary tract symptoms (LUTS) in adult men, significantly affecting their quality of life (QoL) (1). If untreated, BPE can lead to serious complications such as acute urinary retention, hydronephrosis, and acute kidney injury (2).

International guidelines recommend lifestyle changes and pharmacological therapies as initial management for male LUTS (3). Surgical options may be indicated when pharmacotherapy fails or is not tolerated (4). However, these treatments often impact sexual function, particularly ejaculatory function, leading to poor adherence or discontinuation, mostly in young patients who want to preserve antegrade ejaculation (5). Therefore, improvements in minimally invasive and endoscopic methods for BPE have expanded therapeutic options, to minimize side effects and increase treatment efficacy (6).

Endoscopic laser treatments have made significant advances, proving to be effective, but still with significant adverse events and complications, such as retrograde ejaculation (7). Minimally invasive surgical therapies (MISTs) offer faster recovery and effective relief from LUTS with minimal side effects (8). Nevertheless, these newer methods generally have inferior functional results compared to traditional transurethral treatments (9).

In this context, transperineal laser ablation of the prostate (TPLA) has emerged as an alternative option that could maintain ejaculatory function in patients with BPE (10). Recent studies indicate promising perioperative and functional outcomes with TPLA in carefully selected patients with BPE/LUTS (11). This systematic review and meta-analysis aim to assess TPLA efficacy in treating BPE/LUTS and its influence on sexual function.

## MATERIALS AND METHODS

### Study Design

This systematic review and meta-analysis follow the Cochrane Collaboration recommendations and is reported according to the Preferred Reporting Items for Systematic Reviews and Meta-Analyses (PRISMA) statement guideline (Table-S1) (12). The study protocol was registered on June 21st, 2024, in the PROSPERO database, under the identification number CRD42024556034.

### Eligibility Criteria

Inclusion in this meta-analysis was restricted to studies that met the following eligibility criteria: (1) randomized controlled trials (RCTs) or nonrandomized cohorts; (2) transperineal laser ablation of the prostate in treating LUTS decurrent from BPE; and (3) enrollment of male patients older than 18 years with BPE. Additionally, studies were only included if they reported any clinical outcomes of interest, including primary outcome measures related to LUTS relief and side effects. Exclusion criteria were applied to studies with (1) potential overlapping populations; (2) unavailable full text; and (3) publications in non-English languages.

### Search Strategy and Data Extraction

Two authors (I.Z. and M.P.) independently conducted searches on PubMed, Embase, and Cochrane Central Register of Controlled Trials from inception to June 2024, using specific search terms: ‘benign prostatic enlargement’, ‘BPE’, ‘lower urinary tract symptoms’, ‘LUTS’, ‘transperineal laser ablation’, and ‘TPLA’. The complete and detailed search strategy is available in supplementary materials. Reference lists from all included studies were also manually searched for additional studies. Titles and abstracts of all electronic records were screened for potential eligibility. Subsequently, the articles regarded as eligible were retrieved as full texts. Then, any studies that did not report the outcomes of interest or fulfilled inclusion criteria were excluded. Three authors (I.Z., M.P., and M.G) independently extracted data following predefined search criteria and quality assessment. Disagreements were resolved through discussion with a fourth author (R.S.) and, when necessary, by consultation with the senior author (M.A.A).

### Endpoints

Primary endpoints consisted of the International Prostatic Symptoms Score (IPSS) and objective parameters, such as the maximum urinary flow rate (Qmax), prostate volume (PV), and post-void residual (PVR). Secondary endpoints included ejaculatory and erectile function, evaluated by the Male Sexual Health Questionnaire - Ejaculatory Dysfunction (MSHQ-EjD) and the International Index of Erectile Function (IIEF-5); surgical aspects, comprised by operating time and length of stay; and quality of life reported by the IPSS Q8.

### Quality Assessment

Randomized and nonrandomized studies were evaluated using the Cochrane Collaboration's risk-of-bias tools: RoB-2 (13) and ROBINS-I (14), respectively. Two independent authors (T.M. and M.G.) adhered to the Grading of Recommendations, Assessment, Development, and Evaluation (GRADE) handbook guidelines to assess the evidence's certainty level, utilizing categorizations ranging from high to very low (15). Publication bias was investigated by funnel-plot analysis of point estimates according to study weights (16).

## Statistical Analysis

Data was synthesized using a random effects meta-analysis through a restricted maximum likelihood estimator. The random effects model was employed to account for potential clinical, methodological, and statistical heterogeneity since no assumption can be made that there would be no heterogeneity and that the intervention's true effect will be the same in the included studies (16, 17). Continuous endpoints were summarized using mean difference (MD). Additionally, a subgroup analysis was performed to compare outcomes between TPLA and TURP, the conventional standard therapy, from available randomized trials. Statistical significance was established by a 95% confidence interval (CI) and a p-value under 0.05. Evidence of heterogeneity was assessed with the Chi2 test, Tau and Tau2. To avoid misleading interpretation with a pre-determined threshold for I2 statistics, the extent of heterogeneity was evaluated by associating it with the prediction interval (PI) (18, 19). Additionally, a "leave-one-out" sensitivity analysis was performed to identify potential sources of heterogeneity. All statistical analyses were performed in R software version 4.4.1 (R Foundation for Statistical Computing) (20).

For outcome data presented in medians and interquartile ranges (IQRs), we used the most recent calculator to convert them into means and standard deviations (21). Additionally, for the study by Chen et al. (22), which reported outcomes using change scores rather than direct means and SDs, we employed an additional specialized calculator to facilitate the conversion, available at https://www.statstodo.com/CombineMeansSDs.php.

## RESULTS

Study Selection and Baseline Characteristics

**Figure 1:**
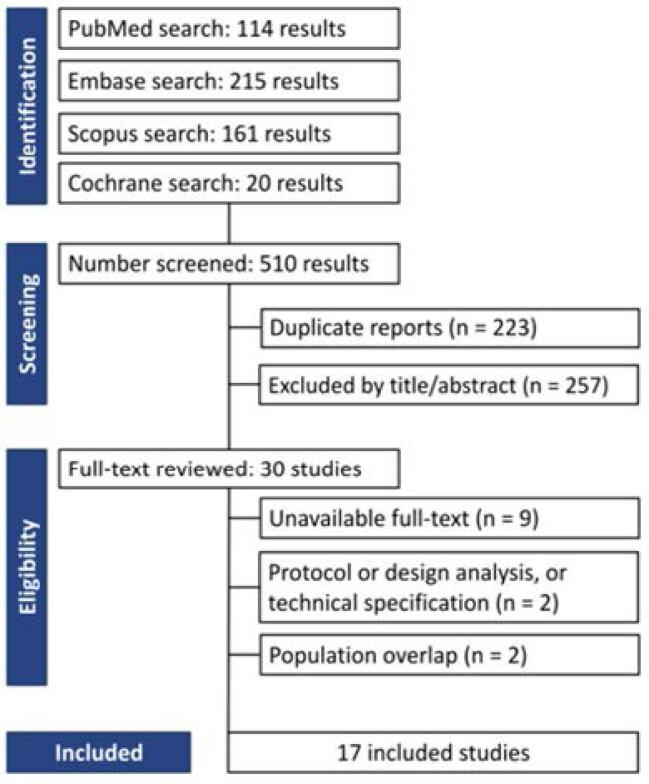
PRISMA flow diagram of study screening and selection. Flow diagram illustrating the process of literature identification, screening, eligibility assessment, and inclusion. Of 510 records initially retrieved, 17 studies met the inclusion criteria and were analyzed in the meta-analysis.

Abbreviations: PRISMA – Preferred Reporting Items for Systematic Reviews and Meta-Analyses.

As reported in Figure-1, the initial search yielded 510 results. After excluding 223 duplicates, 257 articles were excluded based on title and abstract review. Subsequently, 30 articles were fully evaluated. In this comprehensive analysis, 9 articles were excluded due to full-text unavailability, 2 for protocol or design analysis and technical specification, and the last 2 excluded had overlapping populations. In this case, we selected the studies with the larger number of participants or the number of reported outcomes. Finally, 17 studies with 777 patients with BPE were included (22–38). These comprised 3 RCTs and 14 cohort studies, published from 2017 to 2024.

The baseline characteristics of the included studies are shown in Table-1.

**Table 1:** Baseline characteristics of the included studies. Summary of patient demographics, prostate volume, baseline functional parameters, and sexual function indices prior to intervention. Data are presented as mean ± standard deviation (SD) or median (interquartile range, IQR).

Study author/year	Time	N	Age 1 (years)	BMI 1 (kg/m²)	PV 1 (mL) (%)*	PSA 1 (ng/mL)	Qmax 1 (mL/min)	PVR 1 (mL)	IPSS 1	IIEF-5 1	MSHQ-EjD3 1	IPSS-Q8 1
Bertolo, 2023	Baseline	51 (TPLA 26 / TURP 25)	63 (57–70.5)	NA	TPLA 49 (37–65) / TURP 55 (25–88)	TPLA 3.0 (1.1–4.0) / TURP 2.0 (1.2–2.2)	TPLA 10.2 (8.7–12.0) / TURP 10.0 (6.5–11.6)	TPLA 70 (20–100) / TURP 30 (20–70)	TPLA 24.0 (16.0–29.0) / TURP 20 (18.5–24.0)	TPLA 17.0 (15.0–21.0) / TURP 20.0 (16.0–20.5)	TPLA 29.0 (25.0–30.0) / TURP 29.0 (25.5–30.5)	TPLA 5 (3–5) / TURP 4 (3–5)
	1 month	NA	NA	NA	NA	NA	NA	NA	NA	18 (14–23) / 19 (18–23)	29.0 (25.0–30.0) / 20.0 (10.0–25.0)	NA
	6 months	NA	NA	NA	NA	NA	15.2 (13.5–18.3) / 26.0 (22.0–48.0)	0 (0–5) / 0 (0–20)	11 (8–15) / 8 (3–9)	NA	NA	2 (2–4) / 2 (1–2)
Cai, 2021	Baseline	20	73.9 ± 9.2	NA	70.8 ± 23.8	NA	8.5 ± 3.0	78.7 ± 58.8	22.7 ± 5.3	NA	NA	4.9 ± 1.7
	6 months	NA	NA	NA	54.7 ± 20.9	NA	15.2 ± 4.8	30.3 ± 34.2	9.1 ± 3.2	NA	NA	2.3 ± 1.3
Canat, 2023	Baseline	50 (TPLA 25 / TURP 25)	65.58 ± 6.59	TPLA 27.85 ± 2.12 / TURP 27.93 ± 2.27	TPLA 66.77 ± 25.28 / TURP 63.63 ± 21.10	TPLA 4.79 ± 4.63 / TURP 3.71 ± 3.26	TPLA 8.73 ± 3.77 / TURP 8.32 ± 3.54	TPLA 125 ± 68.50 / TURP 139.4 ± 58.73	TPLA 20.14 ± 6.02 / TURP 21.17 ± 4.33	TPLA 14.84 ± 3.93 / TURP 14.17 ± 4.09	TPLA 10.75 ± 2.42 / TURP 11.07 ± 1.87	TPLA 4.75 ± 0.75 / TURP 4.69 ± 0.75
	12 months	NA	NA	NA	TPLA 47.32 ± 13.59 / TURP NA	NA	TPLA 14.26 ± 3.73 / TURP 21.37 ± 6.04	TPLA 46.88 ± 32.40 / TURP 49.13 ± 31.54	TPLA 10.14 ± 3.21 / TURP 10.95 ± 4.33	TPLA 14.68 ± 3.92 / TURP 13.44 ± 4.53	TPLA 10.33 ± 2.31 / TURP 5.93±4.01	TPLA 1.50 ± 0.90 / TURP 1.31 ± 0.75
Chen, 2023	Baseline	51 (TPLA 25 / TURP 26)	69.27 ± 9.67	TPLA 24.38 ± 2.91 / TURP 23.18 ± 2.66	TPLA 60.48 ± 21.1 / TURP 65.19 ± 21.1	TPLA 3.63 ± 1.73 / TURP 3.32 ± 1.84	TPLA 77.14 ± 6.11 / TURP 75 ± 6.27	TPLA 92.5 ± 73,282.5 / TURP 104 ± 62,104	TPLA 23.14 ± 5.38 / TURP 21.40 ± 4.15	TPLA 9.86 ± 4.31 / TURP 10.12 ± 4.28	6.09 ± 3.84 / 6.41 ± 2.28	4.57 ± 0.65 / 4.25 ± 0.58
	3 months^2^				TPLA 73.27 ± 12.94* / TURP 68.59 ± 19.38*	TPLA 0.55 ± 2.02 / TURP −1.56 ± 0.63	TPLA 5.28 ± 4.24 / TURP 16.33 ± 9.82	TPLA −39 (-97,-35) / TURP −85 (-140,-25)	TPLA −14.17 ± 6.13 / TURP −13.19±5.86	TPLA −0.27 ± 1.56 / TURP 0.06 ± 0.93	TPLA 0.49 ± 1.08 / TURP −1.52 ± 0.67	TPLA −2.33 ± 0.89 / TURP −2.5 ± 0.97
Destefanis, 2023	Baseline	40	80 (72.5–84)	24 (22–27)	38 (30.5–73)	2.2 (0.8–3.8)	8 (5.5–10)	38 (30.5–73) cc	25 (19–30)	NA	NA	6 (5–6)
	3 months	NA	NA	NA	35 (26–49)	2.3 (1.7–2.7)	12.5 (9.5–14)	30 (0–60)	10.5 (7.5–13)	NA	NA	3 (0–4)
	6 months	NA	NA	NA	34 (28–49)	1.8 (0.9–8)	12 (10–13)	30 (0–60)	8 (6–11.5)	NA	NA	2 (0–4)
Frego, 2021	Baseline	22	61.9 (55–65.5)	27.16 (24.8–28.6)	65 (46.5–81)	2.24 (1.4–4.5)	9 (5–12.5)	60 (25–107.5)	22 (19.5–25.25)	NA	NA	4 (4–5)
	3 months	NA	NA	NA	46(28.4–69)	NA	12 (9–16.5)	39 (10–87.5)	8 (4.5–11)	22 (19.5–24)	NA	1 (0.5–2)
	6 months	NA	NA	NA	42.3 (39.5–59	NA	15 (11.5–20.5	40(16–63)	5(3–8.5)	23 (20.5–24)	NA	1(0–2)
	12 months	NA	NA	NA	41.5 (36.25–55)	NA	20.5 (14.25–23.75)	30 (5–50)	6(4.25–7)	21.5 (17.25–23.75)	NA	1 (1–2)
Kollenburg, 2024	Baseline	20	70.3 ± 7.3	NA	65.5 ± 23.0	5.0 ± 3.3	9.7 ± 3.5	61.8 ± 58.3	21.3 ± 5.2	35.4 ± 23.6	NA	4.9 ± 0.9
Frego, 2021	3 months	NA	NA	NA	NA	NA	12.8 ± 6.1	64.8 ± 70.4	12.8 ± 6.0	36.9 ± 24.8	NA	2.6 ± 1.7
Kollenburg, 2024	6 months	NA	NA	NA	NA	NA	12.1 ± 6.1	74.2 ± 87.4	11.7 ± 5.2	40.5 ± 23.3	NA	1.8 ± 1.0
Lagana, 2023	12 months	NA	NA	NA	63.2 ± 20.6	NA	14.9 ± 6.0	44.2 ± 55.8	10.9 ± 5.5	31.1 ± 24.4	NA	1.9 ± 1.1
Laganà 2023	Baseline	63	72.3 ± 10	30.2 ± 7.1	63.6 ± 29.7	4.82 ± 1.8	8.6 ± 3.5	124.8 ± 115.4	20.8 ± 7.4	NA	NA	4.7 ± 1.4
Manenti, 2021	3 months	NA	NA	NA	45.6 ± 21.8	NA	NA	43.6 ± 53.6	11.0 ± 6.6	NA	NA	1.5 ± 1.2
Pacella, 2019	12 months	NA	NA	NA	NA	2.89 ± 1.2	16.2 ± 4.3	40.6 ± 53.6	8.4 ± 5.9	NA	NA	1.2 ± 0.8
Lo Re, 2024	Baseline	100	66.5 (60–75)	25.9 (23.5–27.6)	50 (40–70)	NA	9.1 (6.9–12)	90 (50–150)	18 (15–23)	NA	6 (2–11)	4 (3–4)
Patelli, 2024	3 months	NA	NA	NA	NA	NA	11 (8.8–14.8)	45 (20–77,5)	10 (6–13)	NA	10 (5–13)	2 (1–3)
Polverino, 2023	6 months	NA	NA	NA	NA	NA	11 (8.5–16.0)	50 (20–90)	10 (5.7–14)	NA	11 (5–14)	2 (1–3)
Rienzo, 2021	12 months	NA	NA	NA	NA	NA	13 (8.5–16.9)	45 (1.2–87.5)	10 (5–16.5)	NA	9 (5–13)	2 (1–3)
Manenti, 2021	Baseline	44	72.1 ± 6.6	NA	102.42 ± 36.3	7.3 ± 1.8	7.6 ± 4.2	138.4 ± 40.8	18.5 ± 5.5	21 ± 4	4.9 ± 3.7	5.8 ± 1.4
	12 months	NA	NA	NA	48.12 ± 19.2	2.1 ± 0.8	16.2 ± 4.9	18.8 ± 8.5	6.2 ± 3.8	22 ± 3	7.7 ± 3.2	2.1 ± 1.1
Minafra, 2023	Baseline	21	63 (55–70)	NA	41.5 (40.0–54.3)	NA	8.8 (7.8–10.8)	70.0 (33–120)	18 (16–21)	17 (15–21)	4 (3–8)	4 (4–5)
	6 months	NA	NA	NA	NA	NA	13.9 (5.0–32.0)	14.0 (0–50)	6 (3–12)	18 (3–25)	9 (15–13)	2 (1–3)
	3 years	NA	NA	NA	35.0 (32.0–38.8)	NA	11.0 (9.0–12.8)	15.0 (0– 25)	12 (10–15)	17 (15–20)	11(7–21)	2 (1–2)
Pacella, 2019	Baseline	160	69.8 ± 9.6	NA	75.0 ± 32.4	NA	8.0 ± 3.8	89.5 ± 84.6	22.5 ± 5.1	NA	NA	4.5 ± 1.1
	6 months	NA	NA	NA	60.3 ± 24.5	NA	14.3 ± 3.9	27.2 ± 44.5	7.7 ± 3.3	NA	NA	1.8 ± 1.0
	12 months	83	NA	NA	58.8 ± 22.9	NA	15.0 ± 4.0	17.8 ± 51.0	7.0 ± 2.9	NA	NA	1.6 ± 0.9
Patelli, 2017	Baseline	18	71.7 ± 9.4	NA	69.8 ± 39.9	NA	7.6 ± 2.7	199.9 ± 147.3	21.9 ± 6.2	NA	NA	4.7 ± 0.6
	3 months	NA	NA	NA	54.8 ± 29.8	NA	13.3 ± 76.2	81.5 ± 97.8	10.7 ± 4.7	NA	NA	2.1 ± 1.2
Patelli, 2024	Baseline	40	65.1 ± 8.3	NA	66 (48.5–86.5)	NA	9.8 ± 6.2	108 (38–178)	23 (19–26)	NA	NA	5 (4–5)
	12 months	NA	NA	NA	46 (36–65)	NA	12.8 ± 7.4	13.5 (0–40.5)	5 (4–9)	NA	NA	1 (0–1)
	24 months	NA	NA	NA	48 (31–84)	NA	10.8 ± 6.9	23 (5–54)	5 (4–10)	NA	NA	1 (0–1)
	36 months	38	NA	NA	49.5 (31–79)	NA	10.4 ± 4.8	21 (5–49)	7 (3–10)	NA	NA	1 (0–2)
Polverino, 2023	Baseline	23	77 (68–84)	24.5 (22–27)	42 (39–70)	NA	NA	NA	NA	NA	NA	4 (3–5)
	12 months	NA	NA	NA	NA	NA	NA	NA	NA	NA	NA	2 (1–3)
Rienzo, 2021	Baseline	21	62 (54–69)	27 (25–28)	40 (40–50)	2.0 ± 1.1	9.2 ± 3.4	81.8 ± 62.6	18.3 ± 3.9	17.8 ± 6.6	5.7 ± 4.5	4.1 ± 1.0
	1 months	NA	NA	NA	NA	3.0 ± 1.9	12.1 ± 6.4	37.4 ± 25.7	12.0 ± 5.6	17.4 ± 5.0	9.6 ± 4.1	2.4 ± 1,6
	3 months	NA	NA	NA	NA	1.7 ± 0.8	13.3 ± 6.7	18.7 ± 21.2	8.3 ± 3.8	17.7 ± 6.7	6.8 ± 3.5	1.4 ± 0.9
	6 months	NA	NA	NA	NA	1.7 ± 0.8	13.9 ± 6.2	14.0 ± 17.7	6.1 ± 2.6	18.3 ± 5.7	8.6 ± 3.1	1.7 ± 0.8
Sessa, 2022	Baseline	38	71.5 (63.5–79)	25 (22–29)	46 (38–71)	1.86 (0.56–2.76)	9.1 (8–11.5)	100 (70–150)	20 (16–25)	15 (7–24)	6 (2–10)	4 (3–5)
	1 month	NA	NA	NA	NA	NA	10.6 (9–13.6)	55 (32.5–97.5)	15 (12–20)	16 (8–21)	7 (6–10)	3 (2–4)
	3 months	NA	NA	NA	NA	NA	11 (9.4– 13.6)	50 (35–95)	11 (9–16)	18 (8–24)	8 (7–11)	1 (1–3)

NA, not available; BMI, body mass index; IIEF, International Index of Erectile Functions; IPSS, International Prostatic Symptoms Score; MSHQ-EJD, Male Sexual Health Questionnaire-Ejaculatory Dysfunction; PSA, prostate-specific antigen; PV, prostate volume; PVR, Post-Void Residual; Qmax, Maximum urinary flow; IPSS-Q8, International Prostatic Symptoms Score - question 8; TPLA, Transperineal Prostate laser ablation; TURP, transurethral resection of the prostate.

1 Mean (Standard Deviation) or median (IQR).

2 Variation from the baseline to 3 months.

### Operative and Perioperative Aspects

All the patients were placed in lithotomy position. An 18Fr three-way vesical catheter was placed and continuous saline irrigation for urethral cooling was applied. The procedure was performed under transrectal ultrasound (TRUS) guidance. The use of a multi-channel needle applicator with a dedicated software display grid overlapping the ultrasound images could also aid the procedure (23, 37). Local anesthesia was administered in 16 studies (22, 24–38), with concurrent conscious sedation or optimal sedation used in 13 studies (25–30, 32–38). One study performed standard spinal anesthesia (23).

TPLA was performed using EchoLaser (SoracteLite) and Asclepion. The diode laser generator with four independent channels, provided by Elesta, was employed for all procedures, except Chen et al. 2023 (22), where Asclepion Laser Technologies was provided. A 21G trocar needle was used to accommodate the 300-μm flat-tip optical fiber and a continuous mode with a wavelength of 1064 nm was employed. Lo Re, Sessa, De Rienzo, and Manenti (30, 31, 37, 38) initially set the power at a higher level (5 W, 4.5 W, and 5 W, respectively) and then reduced it after 1-2 minutes, while others used a fixed power setting of 3 W. The power deployed by Chen varied from 3 to 5 W (22).

The energy setting for the single fiber was 1800 J, except for Patelli and Sessa (34, 38), who reported settings ranging from 1200 to 1800 J. Up to three fibers per lobe were used with simultaneous laser emission, depending on prostate volume and surgeon preference. A second ablation cycle, called pull-back, was executed in larger prostates. This involved retracting the fiber 10 mm along its trajectory to deliver an additional 1200–1800J (22, 24, 26–29, 31–35, 37, 38).

Ten studies used antibiotic prophylaxis (26–29, 31, 33–35, 37, 38). At the end of the treatment, four studies utilized dexamethasone to reduce edema and inflammatory reactions, (6, 25, 27, 33) while two studies prescribed prednisone (31, 37). Chen et al. and Sessa et al. applied one dose of dexamethasone and methylprednisolone intravenously after treatment, respectively (22, 38). The mean procedural time ranged from 16 to 60.9 minutes (24, 25). Additionally, the mean length of stay ranged from 1.5 hours to 2.5 days (22, 24), while the catheterization period ranged from 4 to 22.8 days (23, 35).

Table-S2 summarizes the technical parameters of all selected studies.

### Inclusion and Exclusion Criteria of the Included Studies

The inclusion and exclusion criteria differed between the studies. Eligible studies normally included patients over 18 years old with a PV ranging from 30 to 100 mL, measured by TRUS or magnetic resonance imaging (MRI), who were candidates for treatment with TPLA. The usual inclusion criteria also involved LUTS with an IPSS of 8 or more, a Qmax of 15 mL/s or less, or a PVR of 50 to 400 mL.

Ten studies reported the pharmacological treatments used for BPE (23, 26–31, 35, 37, 38). One study (31) focused exclusively on patients using combination therapy, while another did not describe the pharmacological treatment (35).

Common exclusion criteria included previous procedures on the urethra or prostate, prostate-specific agent levels higher than 4 ng/mL or suspected prostate cancer, a history of urethral stricture, neurological diseases, allergies to ultrasound contrast, underactive detrusor, bladder cancer, anterior prostatic abscess, acute or chronic prostatitis, active urinary tract infection, gland volume greater than 100 mL, bladder stones and active hematuria. Table-S3 provides a detailed list of these conditions. Some studies did not contraindicate the treatment for patients with a median lobe / intravesical prostatic protrusion (IPP) (27, 28, 33, 34, 37) or taking anticoagulants or antiplatelet agents (23, 25–27, 30, 33, 36–38). However, Minafra et al. (32) reported that a predictive factor for treatment failure in their cohort was the presence of the median lobe/IPP.

### Functional Outcomes by Follow-up Time

**Figure 2:**
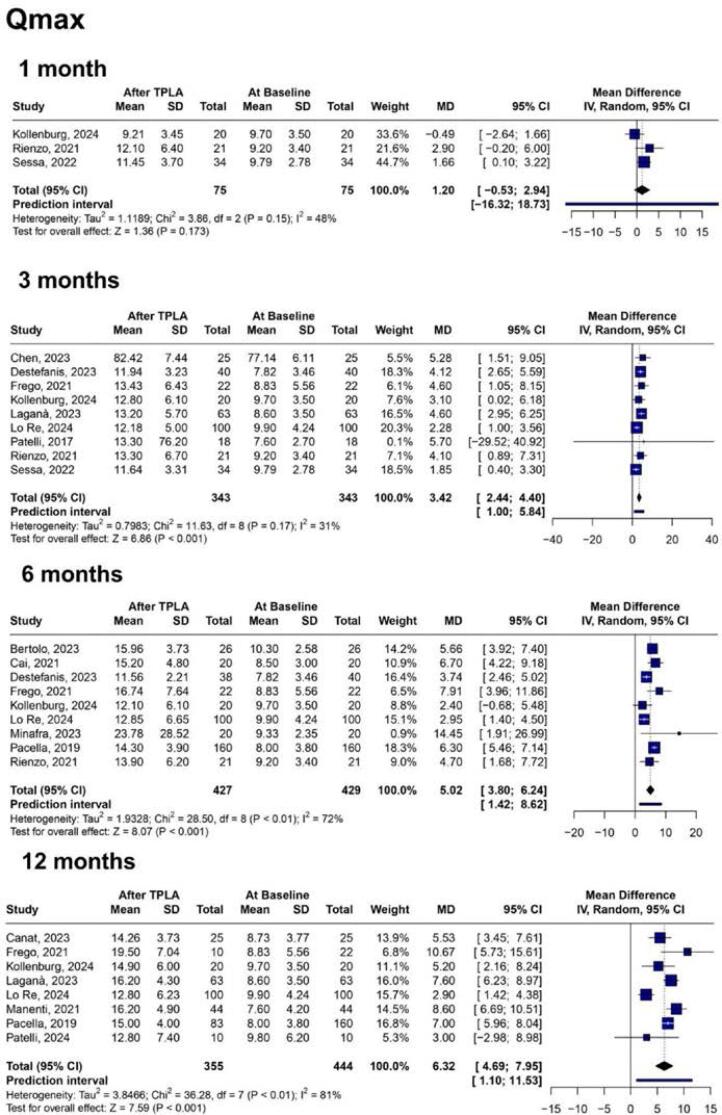
Forest plots of changes in Qmax at different follow-up intervals after TPLA. TPLA produced a consistent and statistically significant improvement in urinary flow over time, reflecting enhanced bladder emptying capacity.

**Figure 3:**
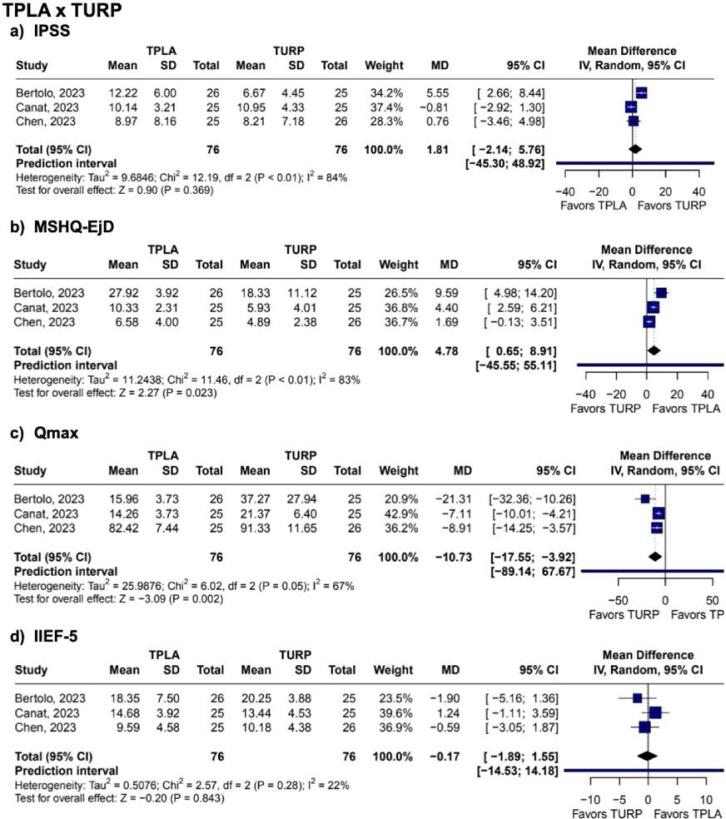
Forest plots of changes in IPSS at different follow-up intervals after TPLA. Pooled analysis demonstrates progressive and significant reduction in IPSS from baseline to 12 months, indicating sustained symptomatic relief in LUTS.

Abbreviations: IPSS – International Prostate Symptom Score; CI – Confidence Interval; MD – Mean Difference; LUTS – Lower Urinary Tract Symptoms; TPLA – Transperineal Prostate Laser Ablation.

In our pooled analysis, improvement in Qmax was observed after three months of treatment (MD 3.42 mL/s; 95% CI 2.44 to 4.40; p<0.001; I2 = 31%. Figure-2). Within six and twelve months, Qmax increased progressively (MD 5.02 mL/s; 95% CI: 3.80 to 6.24; p<0.001; I2 = 72%, and MD 6.32 mL/s; 95% CI 4.69 to 7.95; p<0.001; I2 = 81%. Figure-2). TPLA was associated with a significant decrease in IPSS as of one-month follow-up (1 month: MD −4.48; 95% CI −6.92 to −2.03; p<0.001; I2 = 41%. 3 months: MD −11.11; 95% CI −12.72 to −9.51; p<0.001; I2 = 66%. 6 months: MD −12.46; 95% CI −14.25 to −10.66; p<0.001; I2 = 82%; 12 months: MD −12.62; 95% CI −14.87 to −10.37; p<0.001; I2 = 90%. Figure-3; Figure S13 and S14). Reduction in prostate volume was observed within twelve months (MD −21.23 cm³; 95% CI −32.65 to −9.81; p<0.001; I2 = 84%. Figure-S1). PVR also decreased over twelve months (3 months: MD −46.09 mL; 95% CI −65.66 to −26.51; p < 0.001; I2 = 62%. 6 months: MD −48.30 mL; 95% CI −60.53 to −36.07; p < 0.001; I2 = 57%. 12 months: MD −73.24 mL; 95% CI −96.91 to −49.57; p<0.001; I2 = 89%. Figure-S2). However, in the first month after the surgery, it had no statistically significant change (MD −28.78 mL; 95% CI −57.91 to 0.35; p = 0.053; I2 = 55%. Figure-S2). TPLA was associated with better quality of life by decreasing the IPSS Q8 score in six, twelve, and thirty-six months (MD −2.60; 95% CI −2.99 to −2.22; p < 0.001; I2 = 70%. MD −3.07; 95% CI −3.51 to −2.62; p < 0.001; I2 = 89%. MD −3.19; 95% CI −4.06 to −2.32; p < 0.001; I2 = 83%. Figure-S3).

### Sexual Function by Follow-up Time

Eight studies analyzed ejaculatory dysfunction by MSHQ-EjD. At one-month follow-up, there was no statistically significant change (MD 1.91; 95% CI −0.29 to 4.10; p = 0.089; I2 = 62%. Figure-S4). After three and six months, there was a significant improvement in ejaculatory function (MD 2.01; 95% CI 0.71 to 3.31; p = 0.002; I2 = 32%, and MD 3.28; 95% CI 1.93 to 4.6; p < 0.001; I2 = 0%. Figure-S4). After twelve months, the ejaculatory function remained stable compared to baseline (MD 1.64; 95% CI −0.47 to 3.75; p = 0.127; I2 = 85%. Figure-S4). The IIEF-5 was performed in nine studies to evaluate erectile function. There was no significant statistical alteration in erectile function after the surgery during twelve months (MD 0.54; 95% CI −0.62 to 1.69; p = 0.363; I2 = 0%. Fugire-S5).

### Comparative Analysis Studies (TPLA x TURP)

**Figure 4:**
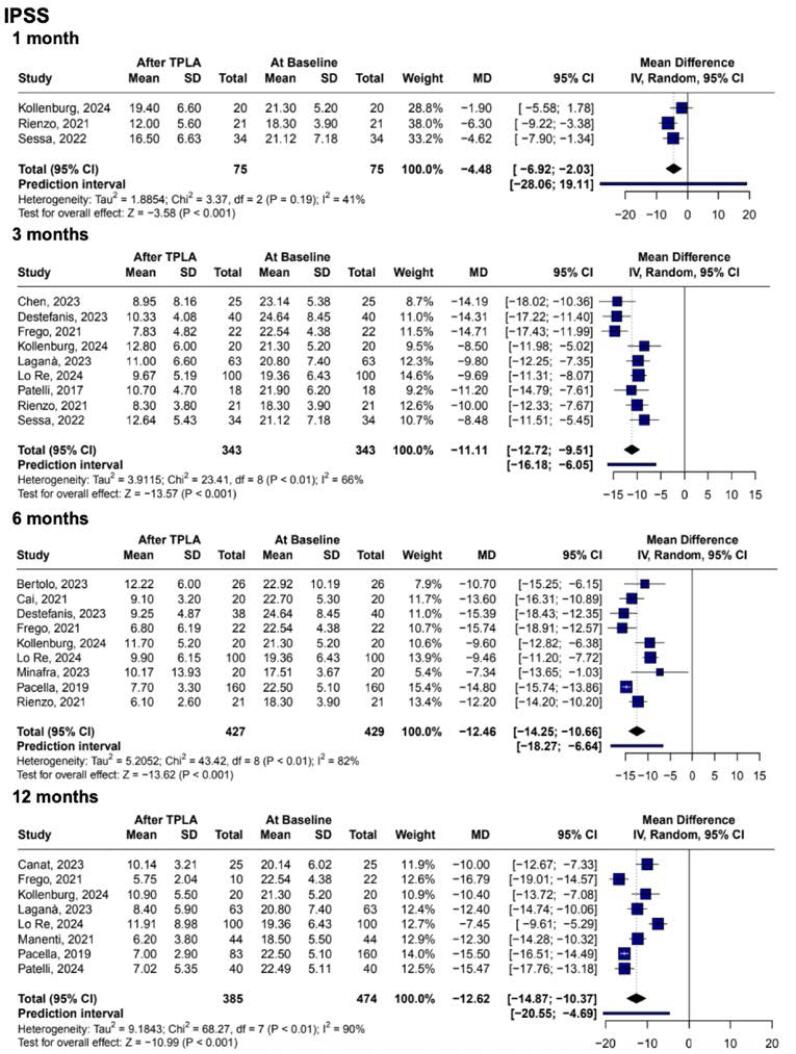
Comparative forest plots between TPLA and transurethral resection of the prostate (TURP): (a) IPSS; (b) Ejaculatory function (MSHQ-EjD); (c) Maximum urinary flow rate (Qmax); (d) Erectile function (IIEF-5). There was no significant difference in LUTS relief (IPSS), and TPLA preserved ejaculatory function (MSHQ-EjD). TURP achieved greater improvement in urinary flow (Qmax), while erectile function (IIEF-5) remained comparable between techniques.

Abbreviations: TPLA – Transperineal Prostate Laser Ablation; TURP – Transurethral Resection of the Prostate; IPSS – International Prostate Symptom Score; MSHQ-EjD – Male Sexual Health Questionnaire for Ejaculatory Dysfunction; Qmax – Maximum Urinary Flow Rate; IIEF-5 – International Index of Erectile Function; CI – Confidence Interval; MD – Mean Difference.

In our subgroup analysis of RCTs comparing TPLA against TURP, there was no significant difference in the treatment of LUTS, as assessed by the IPSS (MD 1.81; 95% CI −2.14 to 5.76; p = 0.369; I2 = 84%. Figure-4A). Additionally, TPLA was demonstrated to be more effective in preserving ejaculatory function, as measured by the MSHQ-EjD (MD 4.78; 95% CI 0.65 to 8.91; p = 0.023; I2 = 83%. Figure-4B). Conversely, TURP was more effective in improving the Qmax (MD −10.73mL/s; 95% CI −17.55 to −3.92; p = 0.002; I2 = 67%. Figure-4C). IIEIF-5 did not differ and showed no statistically significant difference between the procedures (MD −0.17; 95% CI −1.89 to 1.55; p = 0.843; I2 = 22%. Figure-4D). TPLA presented lower operating time and length of stay compared to TURP (MD −43.46min; 95% CI −47.26 to −39.65; p < 0.001; I2 = 4%, and MD −0.54 days; 95% CI −0.73 to −0.35; p < 0.001; I2 = 0%. Figure-S6 A and B)

### Leave-one-out analysis

To explore heterogeneity, a sensitivity analysis was performed to detect studies contributing to the I^2^ value. In the Qmax and PVR outcomes at one month, after omitting the study by Kollenburg, a significant result was found, with heterogeneity reduced to zero. Additionally, omitting Kollenburg et from IPSS outcome at 1 month follow up, the heterogeneity reduced to zero. Still in the first month, regarding the IIEF outcome, after omitting Kollenburg, the heterogeneity resulted in zero, and regardless of the excluded study, no significance was observed. At 12 months of follow up, Canat et al. significantly contribute to the high heterogeneity in the MSHQ-EjD outcome. Excluding this study, TPLA demonstrated to significantly improve the ejaculatory function by 12 months (MD 2.75; CI 95% 1.63 to 3.86; I2 = 0%). The sensitivity analysis of the single arm outcomes by follow up is illustrated in Figures S7, S8, and S9. The leave-one-out sensitivity analysis of the RCTs did not identify a study for the possible source of heterogeneity for most of the outcomes. However, omitting Bertolo et al. of the Qmax analysis, the heterogeneity reduced to zero, and omitting Chen et al. from the PVR outcome, the heterogeneity, also, was zero, but the results in both outcomes were still the same. The sensitivity analysis of the RCTs is shown in Figure-S10.

### Complications

Fourteen studies described the type and the number of complications (22–24, 26–34, 37, 38), and eight classified it according to the Clavien–Dindo system (26–30, 33, 36, 37). Sessa et al. (38) did not describe postoperative complications or sequelae in detail; nevertheless, they specified that no Clavien-Dindo grade ≥2 complications were experienced. Acute urinary retention, urinary tract infection, hematuria, and prostatic abscess were the most frequent complications. According to Chen et al. (22), TPLA had fewer complications than TURP (16% vs. 19.23%). Most of TPLA complications were Clavien-Dindo grade I and II. Table-S4 specifies all the reported complications.

### Risk of Bias and Certainty of Evidence

The overall risk of bias for most of the non-randomized studies was moderate (Figure-S11), and low for the randomized studies (Figure-S12). The full GRADE assessment of the certainty of evidence is available in the supplementary materials (Table-S5).

## DISCUSSION

The novel therapeutic options for BPE aim to treat non-neurogenic LUTS and avoid sexual side effects, which are a major source of dissatisfaction for men undergoing treatments for BPE. Therefore, the sexual side effects should be carefully considered, and the patient should be properly counseled before starting medical or surgical therapies. MISTs are becoming a new promise, especially with the concern of preserving sexual function and improving urodynamics parameters.

In this systematic review and meta-analysis of 17 studies and 777 patients, TPLA was assessed as a single-arm intervention and against the conventional TURP strategy. Our analysis demonstrated that TPLA was able to decrease IPSS and prostate volume from baseline while increasing the maximum urinary flow rate. Concerning the ejaculatory function, evaluated by the MSHQ-EjD, TPLA did not impose a negative effect. No changes were observed in the erectile function measured by the IIEF-5. In addition, TPLA was associated with a shorter operating time and length of stay than TURP. According to Chen et al., there was a minimum per-protocol hospitalization time in the TPLA group of up to 2.5 days. However, there was a benefit in terms of short hospital stays in the studies evaluating the new technology in general, as evidenced in the comparison of TPLA versus TURP in the RCTs (22).

A usual indication for the surgical treatment of BPE is moderate or severe voiding symptoms refractory to drug therapy. Although TURP has remained the gold standard due to its well-established technique and efficacy, it has been linked with numerous complications, (39) while MISTs are generally associated with fewer adverse events. (6) However, despite the American Urological Association (AUA) and European Association of Urology (EAU) guidelines on non-neurogenic male LUTS included MISTs as new therapeutic approaches for selected patients, the recommendations are still low to moderate in strength as they await more robust data (3).

Several trials have evaluated different MISTs interventions as alternatives to TURP, observing favorable outcomes (22, 23, 25). Recent data from a network meta-analysis of RCTs comparing new MISTs with standard surgical methods demonstrated similar symptom improvement profiles in the short and medium term, with less sexual dysfunction. However, the same data indicated that TURP provided greater benefits in increasing Qmax (40). Indeed, our comparative analysis revealed a 10-point difference in post-procedure Qmax favoring TURP. This could be explained by the extent of tissue removal and the immediate effect of TURP compared to the delayed prostatic volume response to TPLA (41). Nevertheless, TPLA has shown a 5-point reduction in Qmax in our analysis, consistent with what is expected from currently available therapies (42). It is worth noting that, while improvements in uroflowmetry parameters are important, patient-centered outcomes are as crucial since LUTS heavily impacts patients’ QoL (43). As such, IPSS has been widely used as a symptom index for BPE and should be repeated after non-invasive and minimally invasive treatments (44). Our pooled analysis revealed an 11-point reduction in IPSS with TPLA treatment, along with no observable difference when compared to TURP, suggesting a similar patient-perceived treatment response.

In regard to sexual function, TPLA did not change erectile function from baseline, as evaluated with the IIEF-5 score (45), nor did it differ when compared to TURP. BPE procedures do not appear to impact erectile function, as stated in a comprehensive review of forty-five RCTs. However, there seems to be lesser risk of retrograde ejaculation with the new MISTs compared to TURP (46). In our pooled analysis, the ejaculatory function, assessed with the MSHQ-EjD form (47), did show a slight improvement from baseline, although we acknowledge that a 1.5-point change may not be clinically relevant. Nevertheless, when compared to TURP, the new treatment was able to preserve ejaculatory function, showing a clear benefit of the procedure. Although the advantages of TPLA over TURP, such as shorter operating time, and preservation of sexual function, are notable, TURP still is more effective in increasing Qmax and other parameters in terms of clinical significance.

Recent data demonstrated that prolonged surgical time may be a modifiable risk factor for complications due to an incidence likelihood of 14% for every additional 30 minutes of surgery, as reported by a meta-analysis of sixty-six studies (48). The impact of surgical time was further assessed by a 10-year analysis of patients undergoing TURP, which demonstrated a significant overall complication rate of 9%, and an increased complication risk as surgical time prolongs (49). In our pooled analysis, TPLA not only reduced operating time but also resulted in a slight decrease in hospitalization time compared to TURP, which could potentially improve safety outcomes and patient willingness to undergo the procedure (23). However, benefits are not limited to patient-related outcomes. Along with technological advancements, shortened operating time and faster recovery may allow these procedures to be performed in an office-based setting (50) and may represent a cost-effective alternative to current standard approaches (30).

This study has limitations. Nearly all included studies were single arm with no comparators, posing a significant limitation to the scope of our analysis. Only three RCTs were included in the analysis, which limited the robustness of the results and affected the certainty of evidence, since most of the included studies were non-randomized and had moderate risk of bias. Furthermore, current literature on TPLA is limited by the short follow-up period (≤ 12 months), unlike other procedures, which have long follow-up periods (51). This limits confidence in durability, retreatment rates, possible late complications, and long-term sexual/functional outcomes; consequently, we moderate the conclusiveness of our statements to reflect these limitations. Moreover, there was variability among procedure techniques. Significant variation in laser settings, procedural protocols, and follow-up durations across studies were noted. Differences in laser power settings, ablation time and a greater number of fibers potentially influence both the efficacy and safety of the procedure, with higher intensities yielding better results, but also increasing the risk of adverse effects. The minimum distance from bladder neck, urethral and between needles, also, had a few variations among studies. This technical and methodological heterogeneity contributes to variability between studies in terms of functional outcomes and complications. Future evidence syntheses should stratify results by key parameters (power/energy settings, fibers per lobe, total energy delivered, and energy density expressed in joules/mL of baseline prostate volume) and evaluate the device platform and perioperative protocols as additional moderators to identify technically optimized and patient-centered protocols, as well as clarify trade-offs between efficacy, ejaculation preservation, and complications. In addition, discrepancies in the duration of follow-up can lead to inconsistent assessments of long-term efficacy, as some benefits or complications may only emerge over time. To increase the clinical applicability of the results, future analyses should consider comparing studies with similar methodologies, grouping them based on key parameters to identify more consistent trends. This approach would provide clinicians with clearer, evidence-based insights to optimize laser treatments and minimize risks.

Although this new technology is being extensively researched, and many recent studies have been published, our study presents significant advances in terms of scope, methodological rigor, and analytical depth. First, among the reviews already published, ours included a larger number of patients (n=777) and studies (17), reflecting a broader and more up-to-date literature search. In addition, we conducted a complete quantitative meta-analysis with the application of random effects models, subgroup analysis (including direct comparisons between TPLA and TURP), assessment of the certainty of evidence via the GRADE approach, and leave-one-out sensitivity analysis to investigate sources of heterogeneity. Another relevant difference was the inclusion of functional data stratified by follow-up time (1, 3, 6, and 12 months), allowing a more detailed view of the clinical evolution of patients. Our work also stood out by presenting quality of life and sexual function data based on validated instruments (MSHQ-EjD and IIEF-5), which reinforces the clinical relevance of the findings. Finally, by strictly following the PRISMA guidelines and registering the protocol in PROSPERO, we ensured transparency and reproducibility. These characteristics consolidate our review as a more comprehensive, current, and methodologically robust contribution to the literature on TPLA in the treatment of LUTS secondary to BPE.

This review provides the most complete quantitative appraisal of TPLA for BPH, integrating symptoms, flow, perioperative, and sexual outcomes with consistent analytic standards (random-effects, sensitivity analyses, GRADE) and head-to-head context versus TURP when available. Beyond summarizing effects, it maps key technical drivers (power/energy, fibers-per-lobe, energy density) that may explain heterogeneity and offers a framework for future studies. These contributions enhance clinical interpretability—particularly around ejaculatory preservation and recovery—while highlighting evidence gaps (nonrandomized designs, short follow-up) that should shape the next generation of trials. Therefore, future randomized trials are advised to be performed in a multicentric fashion with a greater number of patients, comparing other treatment options to increase the generalizability of the findings. Nevertheless, observational studies often include a broader and more diverse population, and allow for a longer follow-up, thus, providing further insights into a real-world clinical setting.

## CONCLUSION

TPLA demonstrated favorable outcomes for LUTS/BPE without a negative impact on sexual function. This minimally invasive treatment was found to have advantages over TURP, such as reduced operative time and shorter hospital stay. The evidence on this new MIST is emerging, but more comparative studies are required to understand the role of this technology, as this study consists mainly of retrospective studies. To date, this is the first meta-analysis to compare TURP and TPLA, and a substantial number of studies published in the literature have been included, although the available evidence is limited.

## Data Availability

All data generated or analysed during this study are included in this published article
