# Supplementary material for: Transperineal Laser Ablation for Treatment of Lower Urinary Tract Symptoms in Benign Prostate Enlargement: A Systematic Review and Meta-analysis
Source: Int Braz J Urol. 2025 May 20;52(2):e20250423. doi: 10.1590/S1677-5538.IBJU.2025.0423 (PMC13124165; doi:10.1590/S1677-5538.IBJU.2025.0423)
Supplement: Supplementary file 1 [file 1677-6119-ibju-52-02-e20250423-suppl1.pdf]

SUPPLEMENTARY MATERIAL

Figure S1. Changes in prostate volume at each follow-up period after TPLA. A progressive and statistically significant decrease in PV was observed up to 12 months, indicating sustained reduction in gland size.

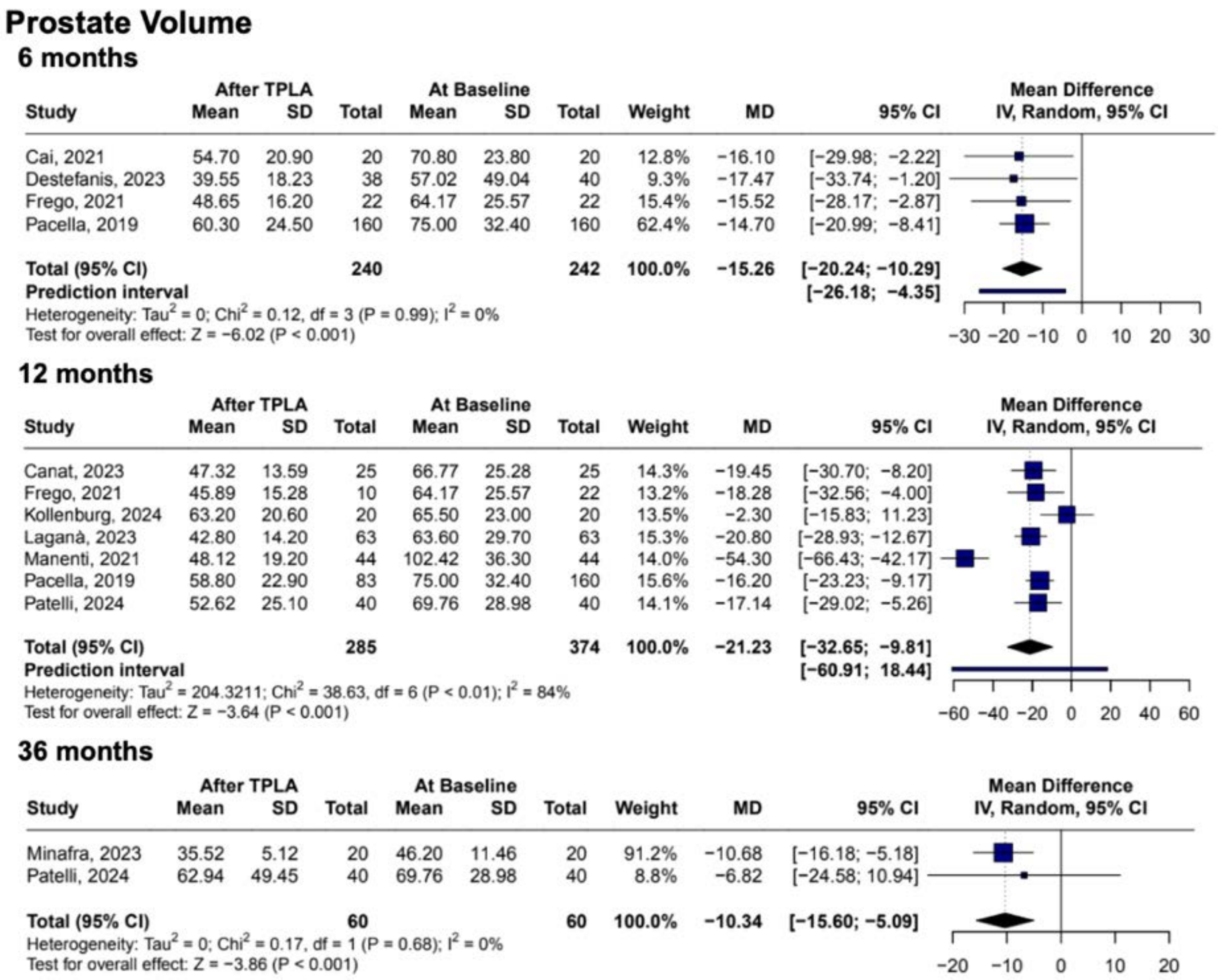

Abbreviations: PV – Prostate Volume; TPLA – Transperineal Prostate Laser Ablation; CI – Confidence Interval; MD – Mean Difference

Figure S2. Changes in post-void residual urine volume at each follow-up period. TPLA significantly reduced PVR from baseline at 3, 6, and 12 months, showing improved bladder emptying over time.

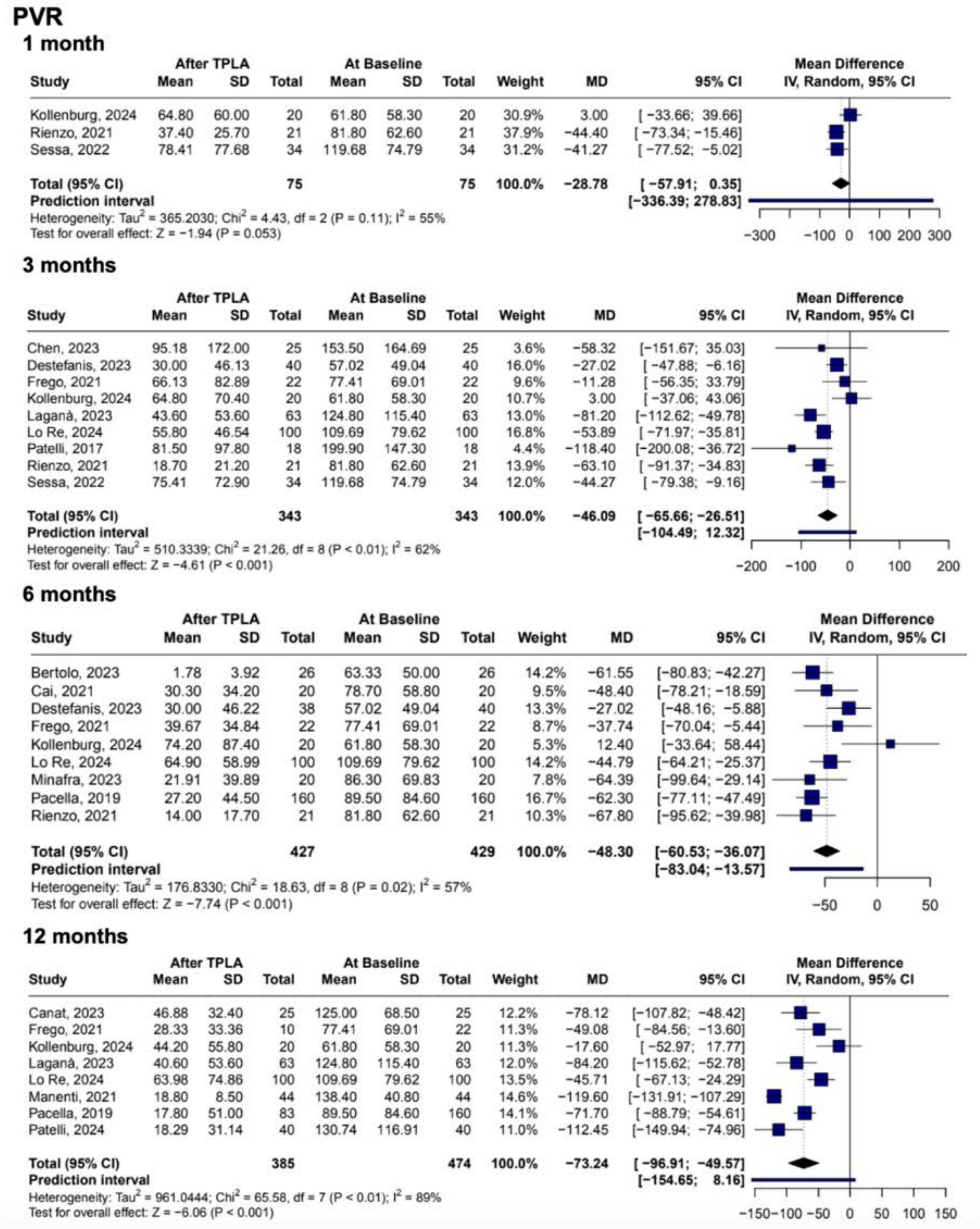

Abbreviations: PVR – Post-Void Residual; TPLA – Transperineal Prostate Laser Ablation; CI – Confidence Interval; MD – Mean Difference.

Figure S3. Changes in quality-of-life IPSS Question 8 following TPLA at each follow-up. The IPSS-Q8 domain, which assesses patients perceived quality of life, improved significantly across all follow-up intervals, reflecting symptom relief and better daily functioning.

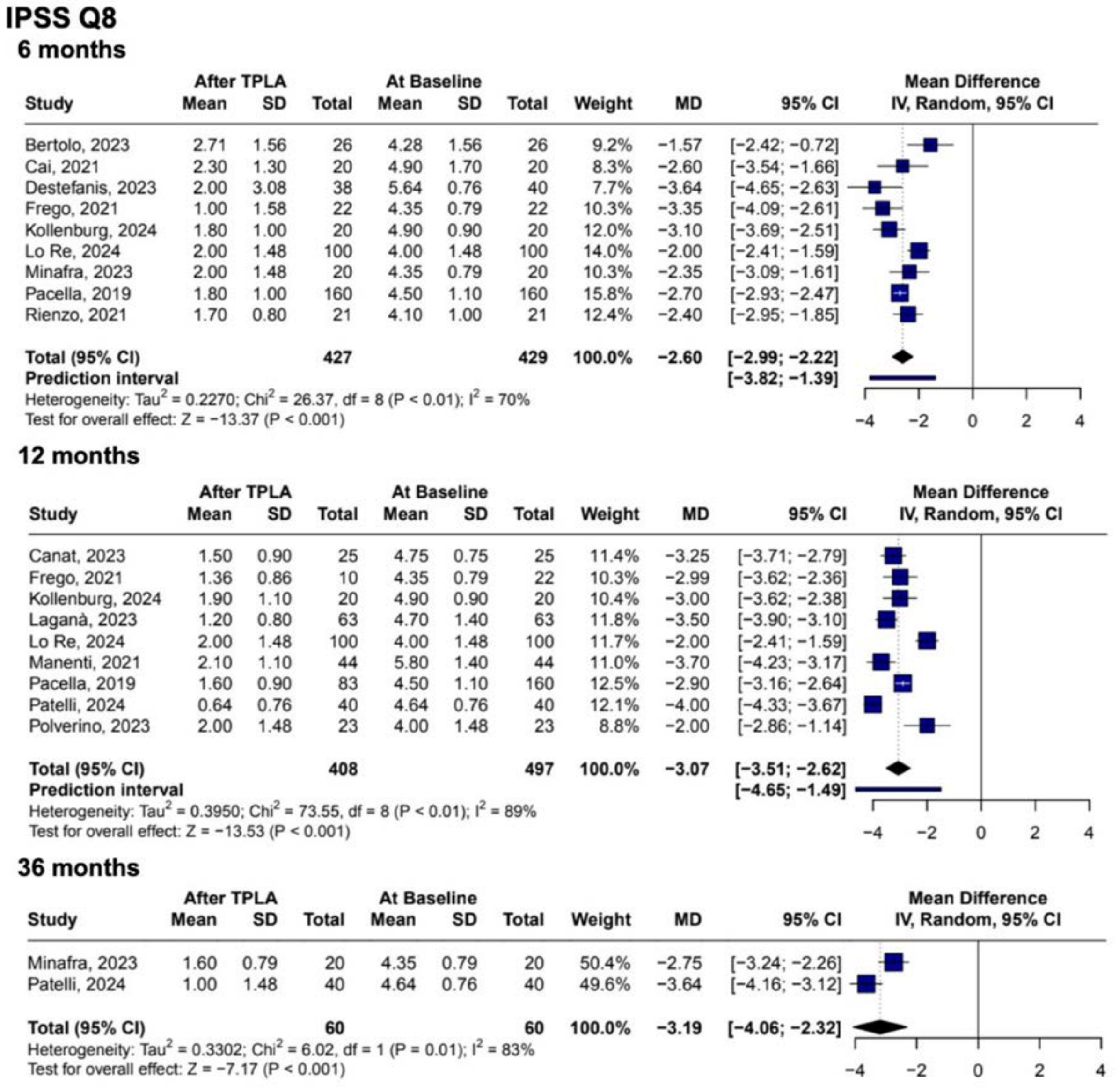

Abbreviations: IPSS – International Prostate Symptom Score; CI – Confidence Interval; MD – Mean Difference; TPLA – Transperineal Prostate Laser Ablation.

Figure S4. Changes in MSHQ-EjD after TPLA. Ejaculatory function showed mild improvement within 3-6 months after the procedure and remained stable thereafter, suggesting preservation of sexual function.

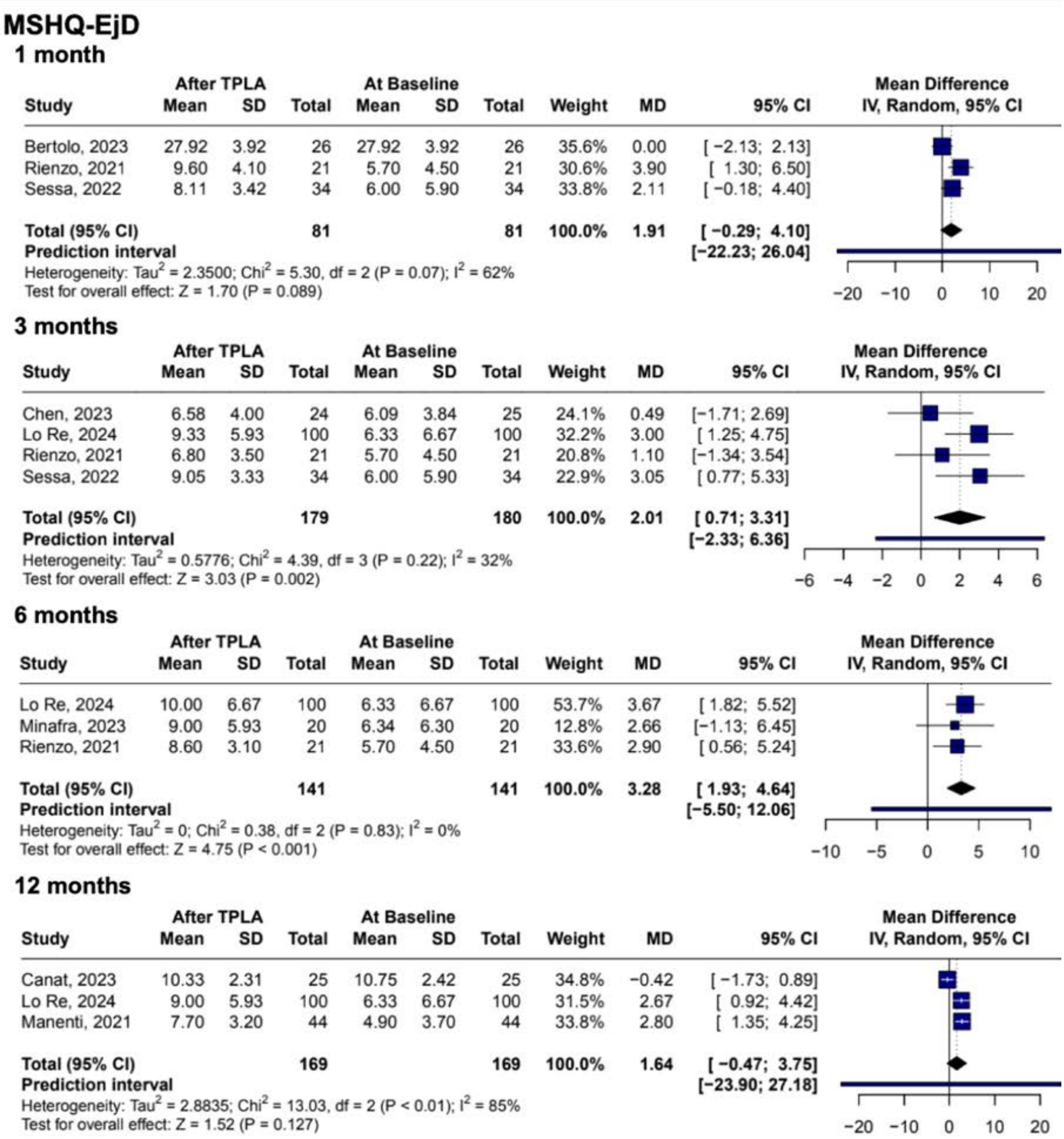

MSHQ-EjD

1 month

Study	After TPLA Mean	After TPLA SD	Total	At Baseline Mean	At Baseline SD	Total	Weight	MD	95% CI
Bertolo, 2023	27.92	3.92	26	27.92	3.92	26	35.6%	0.00	[−2.13; 2.13]
Rienzo, 2021	9.60	4.10	21	5.70	4.50	21	30.6%	3.90	[1.30; 6.50]
Sessa, 2022	8.11	3.42	34	6.00	5.90	34	33.8%	2.11	[−0.18; 4.40]
**Total (95% CI)**			**81**			**81**	**100.0%**	**1.91**	**[−0.29; 4.10]**
**Prediction interval**									**[−22.23; 26.04]**

Heterogeneity: $Tau^2 = 2.3500$; $Chi^2 = 5.30$, df = 2 (P = 0.07); $I^2 = 62\%$
Test for overall effect: Z = 1.70 (P = 0.089)

3 months

Study	After TPLA Mean	After TPLA SD	Total	At Baseline Mean	At Baseline SD	Total	Weight	MD	95% CI
Chen, 2023	6.58	4.00	24	6.09	3.84	25	24.1%	0.49	[−1.71; 2.69]
Lo Re, 2024	9.33	5.93	100	6.33	6.67	100	32.2%	3.00	[1.25; 4.75]
Rienzo, 2021	6.80	3.50	21	5.70	4.50	21	20.8%	1.10	[−1.34; 3.54]
Sessa, 2022	9.05	3.33	34	6.00	5.90	34	22.9%	3.05	[0.77; 5.33]
**Total (95% CI)**			**179**			**180**	**100.0%**	**2.01**	**[0.71; 3.31]**
**Prediction interval**									**[−2.33; 6.36]**

Heterogeneity: $Tau^2 = 0.5776$; $Chi^2 = 4.39$, df = 3 (P = 0.22); $I^2 = 32\%$
Test for overall effect: Z = 3.03 (P = 0.002)

6 months

Study	After TPLA Mean	After TPLA SD	Total	At Baseline Mean	At Baseline SD	Total	Weight	MD	95% CI
Lo Re, 2024	10.00	6.67	100	6.33	6.67	100	53.7%	3.67	[1.82; 5.52]
Minafra, 2023	9.00	5.93	20	6.34	6.30	20	12.8%	2.66	[−1.13; 6.45]
Rienzo, 2021	8.60	3.10	21	5.70	4.50	21	33.6%	2.90	[0.56; 5.24]
**Total (95% CI)**			**141**			**141**	**100.0%**	**3.28**	**[1.93; 4.64]**
**Prediction interval**									**[−5.50; 12.06]**

Heterogeneity: $Tau^2 = 0$; $Chi^2 = 0.38$, df = 2 (P = 0.83); $I^2 = 0\%$
Test for overall effect: Z = 4.75 (P < 0.001)

12 months

Study	After TPLA Mean	After TPLA SD	Total	At Baseline Mean	At Baseline SD	Total	Weight	MD	95% CI
Canat, 2023	10.33	2.31	25	10.75	2.42	25	34.8%	−0.42	[−1.73; 0.89]
Lo Re, 2024	9.00	5.93	100	6.33	6.67	100	31.5%	2.67	[0.92; 4.42]
Manenti, 2021	7.70	3.20	44	4.90	3.70	44	33.8%	2.80	[1.35; 4.25]
**Total (95% CI)**			**169**			**169**	**100.0%**	**1.64**	**[−0.47; 3.75]**
**Prediction interval**									**[−23.90; 27.18]**

Heterogeneity: $Tau^2 = 2.8835$; $Chi^2 = 13.03$, df = 2 (P < 0.01); $I^2 = 85\%$
Test for overall effect: Z = 1.52 (P = 0.127)

Abbreviations: MSHQ-EjD – Male Sexual Health Questionnaire for Ejaculatory Dysfunction; CI – Confidence Interval; MD – Mean Difference; TPLA – Transperineal Prostate Laser Ablation

Figure S5. Changes in IIEF-5 following TPLA. No significant differences were observed at any follow-up, indicating that TPLA does not adversely affect erectile function.

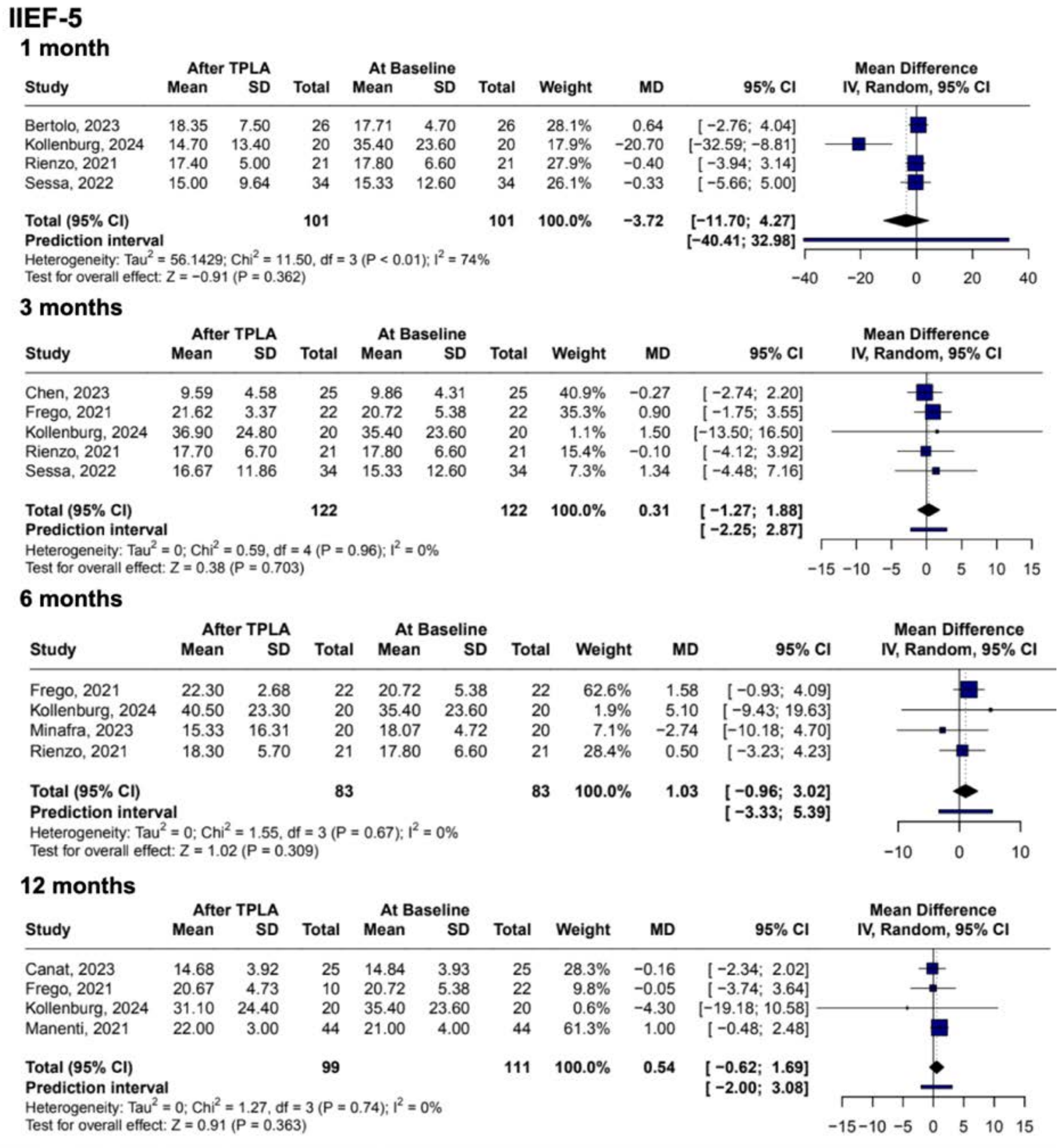

IIEF-5

1 month

Study	After TPLA Mean	After TPLA SD	Total	At Baseline Mean	At Baseline SD	Total	Weight	MD	95% CI
Bertolo, 2023	18.35	7.50	26	17.71	4.70	26	28.1%	0.64	[−2.76; 4.04]
Kollenburg, 2024	14.70	13.40	20	35.40	23.60	20	17.9%	−20.70	[−32.59; −8.81]
Rienzo, 2021	17.40	5.00	21	17.80	6.60	21	27.9%	−0.40	[−3.94; 3.14]
Sessa, 2022	15.00	9.64	34	15.33	12.60	34	26.1%	−0.33	[−5.66; 5.00]
**Total (95% CI)**			**101**			**101**	**100.0%**	**−3.72**	**[−11.70; 4.27]**
**Prediction interval**									**[−40.41; 32.98]**

Heterogeneity: $Tau^2 = 56.1429$; $Chi^2 = 11.50$, df = 3 ($P < 0.01$); $I^2 = 74\%$
Test for overall effect: $Z = -0.91$ ($P = 0.362$)

3 months

Study	After TPLA Mean	After TPLA SD	Total	At Baseline Mean	At Baseline SD	Total	Weight	MD	95% CI
Chen, 2023	9.59	4.58	25	9.86	4.31	25	40.9%	−0.27	[−2.74; 2.20]
Frego, 2021	21.62	3.37	22	20.72	5.38	22	35.3%	0.90	[−1.75; 3.55]
Kollenburg, 2024	36.90	24.80	20	35.40	23.60	20	1.1%	1.50	[−13.50; 16.50]
Rienzo, 2021	17.70	6.70	21	17.80	6.60	21	15.4%	−0.10	[−4.12; 3.92]
Sessa, 2022	16.67	11.86	34	15.33	12.60	34	7.3%	1.34	[−4.48; 7.16]
**Total (95% CI)**			**122**			**122**	**100.0%**	**0.31**	**[−1.27; 1.88]**
**Prediction interval**									**[−2.25; 2.87]**

Heterogeneity: $Tau^2 = 0$; $Chi^2 = 0.59$, df = 4 ($P = 0.96$); $I^2 = 0\%$
Test for overall effect: $Z = 0.38$ ($P = 0.703$)

6 months

Study	After TPLA Mean	After TPLA SD	Total	At Baseline Mean	At Baseline SD	Total	Weight	MD	95% CI
Frego, 2021	22.30	2.68	22	20.72	5.38	22	62.6%	1.58	[−0.93; 4.09]
Kollenburg, 2024	40.50	23.30	20	35.40	23.60	20	1.9%	5.10	[−9.43; 19.63]
Minafra, 2023	15.33	16.31	20	18.07	4.72	20	7.1%	−2.74	[−10.18; 4.70]
Rienzo, 2021	18.30	5.70	21	17.80	6.60	21	28.4%	0.50	[−3.23; 4.23]
**Total (95% CI)**			**83**			**83**	**100.0%**	**1.03**	**[−0.96; 3.02]**
**Prediction interval**									**[−3.33; 5.39]**

Heterogeneity: $Tau^2 = 0$; $Chi^2 = 1.55$, df = 3 ($P = 0.67$); $I^2 = 0\%$
Test for overall effect: $Z = 1.02$ ($P = 0.309$)

12 months

Study	After TPLA Mean	After TPLA SD	Total	At Baseline Mean	At Baseline SD	Total	Weight	MD	95% CI
Canat, 2023	14.68	3.92	25	14.84	3.93	25	28.3%	−0.16	[−2.34; 2.02]
Frego, 2021	20.67	4.73	10	20.72	5.38	22	9.8%	−0.05	[−3.74; 3.64]
Kollenburg, 2024	31.10	24.40	20	35.40	23.60	20	0.6%	−4.30	[−19.18; 10.58]
Manenti, 2021	22.00	3.00	44	21.00	4.00	44	61.3%	1.00	[−0.48; 2.48]
**Total (95% CI)**			**99**			**111**	**100.0%**	**0.54**	**[−0.62; 1.69]**
**Prediction interval**									**[−2.00; 3.08]**

Heterogeneity: $Tau^2 = 0$; $Chi^2 = 1.27$, df = 3 ($P = 0.74$); $I^2 = 0\%$
Test for overall effect: $Z = 0.91$ ($P = 0.363$)

Abbreviations: IIEF-5 – International Index of Erectile Function; CI – Confidence Interval; MD – Mean Difference; TPLA – Transperineal Prostate Laser Ablation.

Figure S6. Comparative analysis between TPLA and TURP: (a) Operating time; (b) Length of hospital stay. Compared to TURP, TPLA demonstrated shorter operative times and reduced hospitalization periods, confirming the minimally invasive nature of the procedure.

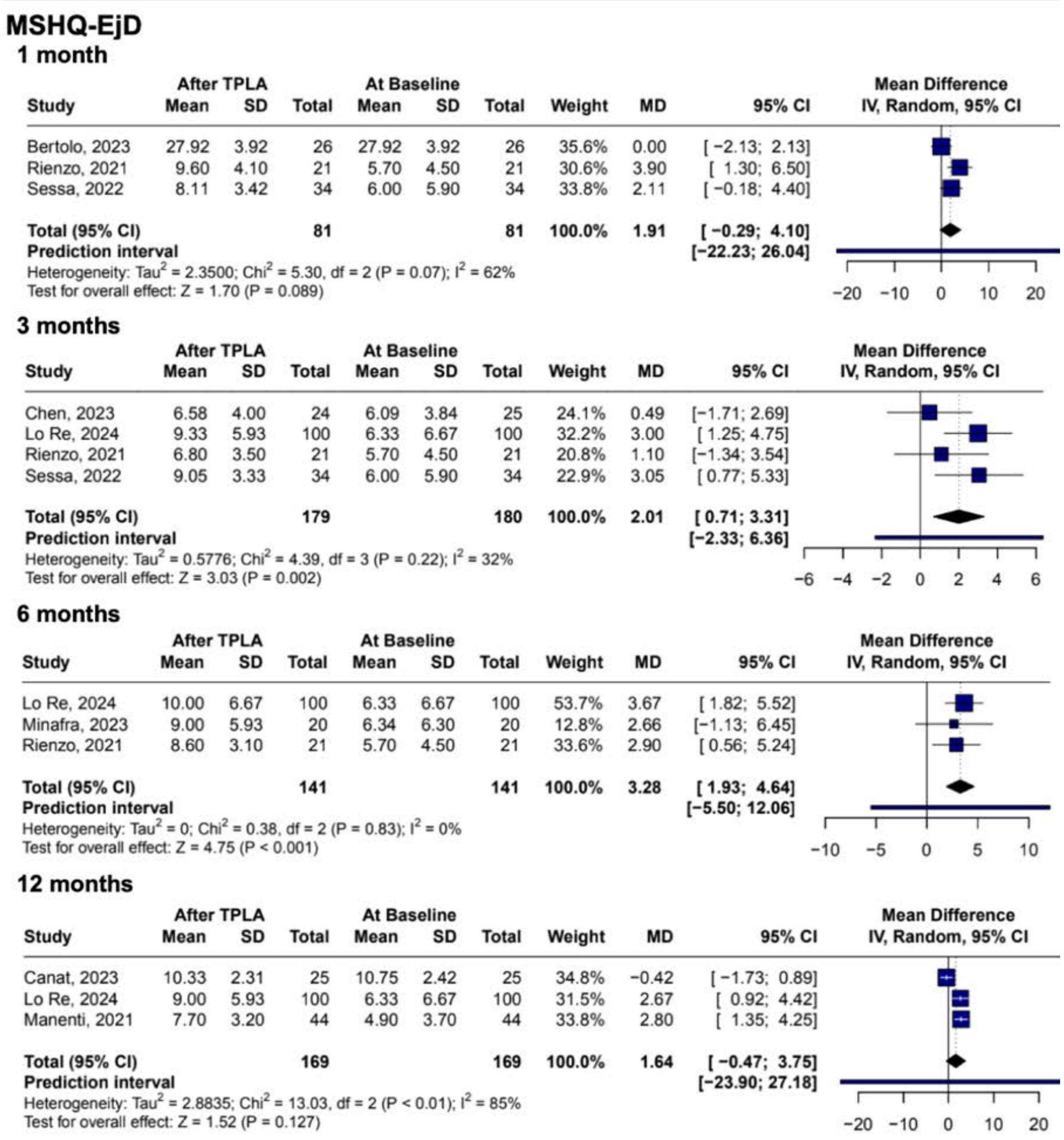

Abbreviations: TPLA – Transperineal Prostate Laser Ablation; TURP – Transurethral Resection of the Prostate; CI – Confidence Interval; MD – Mean Difference.

Figure S7. Risk of bias assessment for non-randomized studies using the ROBINS-I tool. Most studies were classified as having moderate risk of bias, primarily due to non-randomized design and potential confounding.

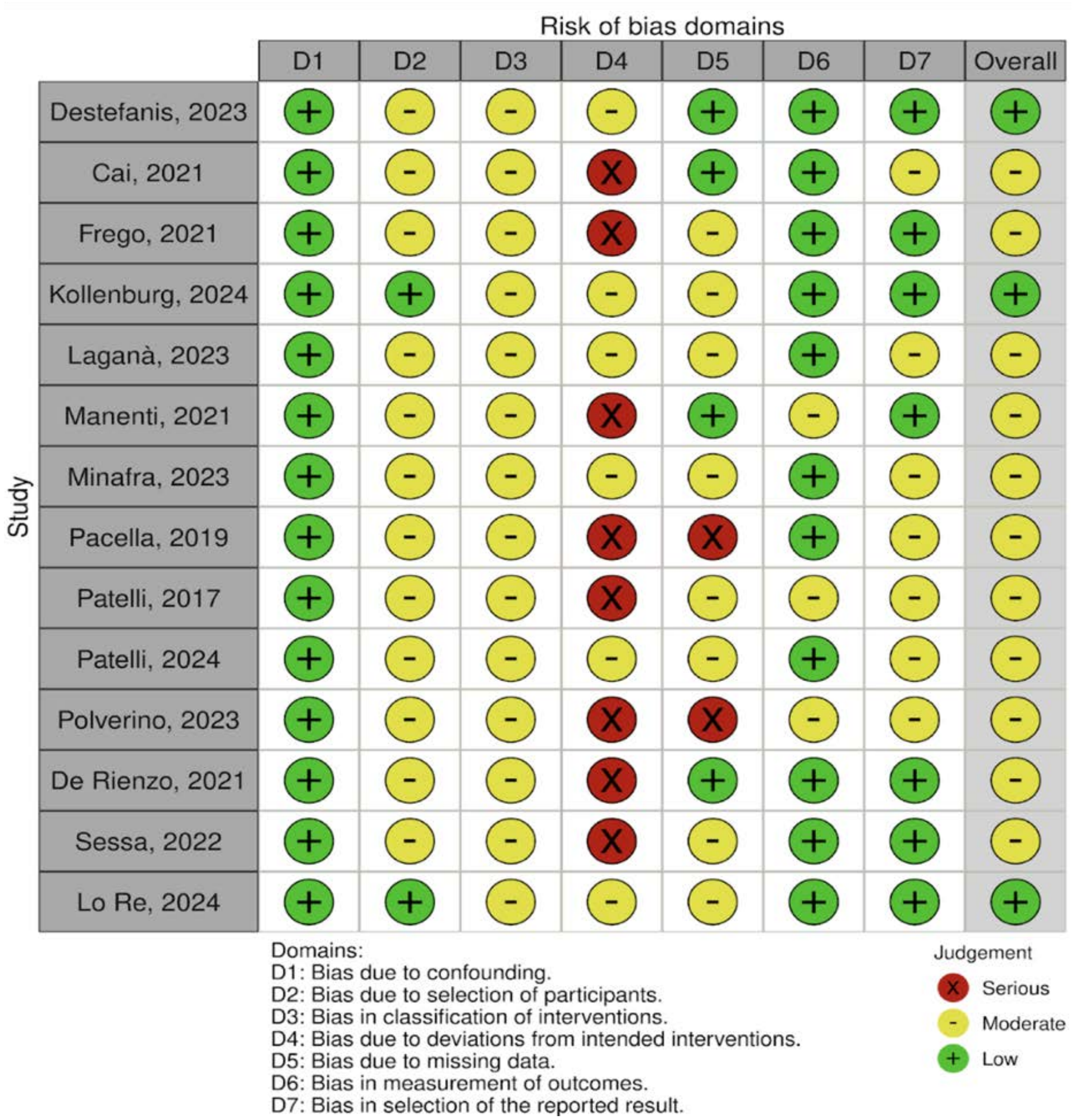

Abbreviations: ROBINS-I – Risk Of Bias In Non-randomized Studies of Interventions

Figure S8. Risk of bias assessment for randomized controlled trials using the RoB 2 tool. All included randomized trials demonstrated low risk of bias across major domains, supporting the robustness of comparative findings.

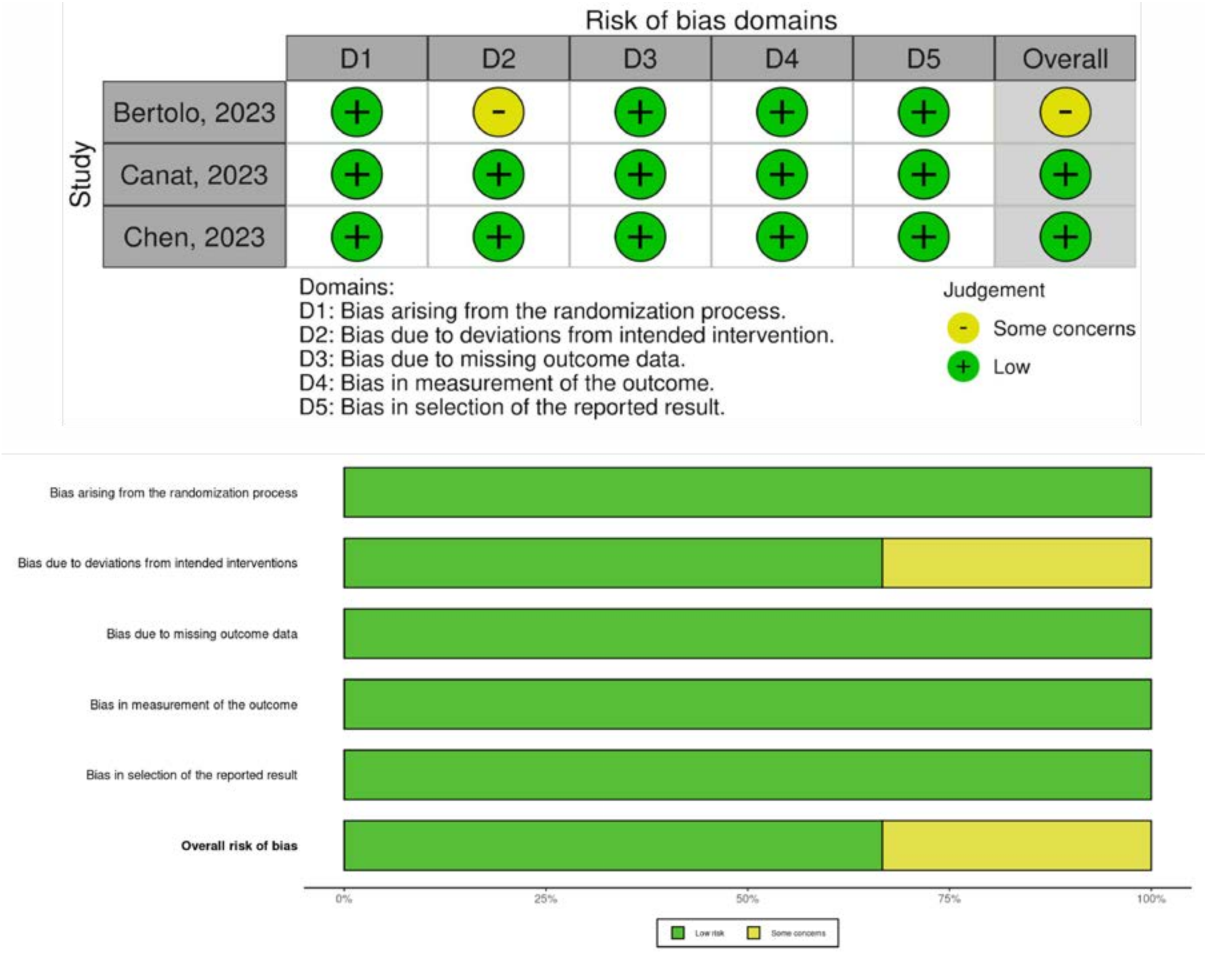

Abbreviations: RoB 2 – Revised Cochrane Risk of Bias tool for Randomized Trials.

Figure S9. Funnel plot for IPSS change at 3 months after TPLA. The scatter appears approximately symmetric around the pooled mean difference, with no prominent visual asymmetry. Given the limited number of studies at this time point, we cannot exclude small-study effects; observed dispersion is compatible with between-study heterogeneity in technique and follow-up.

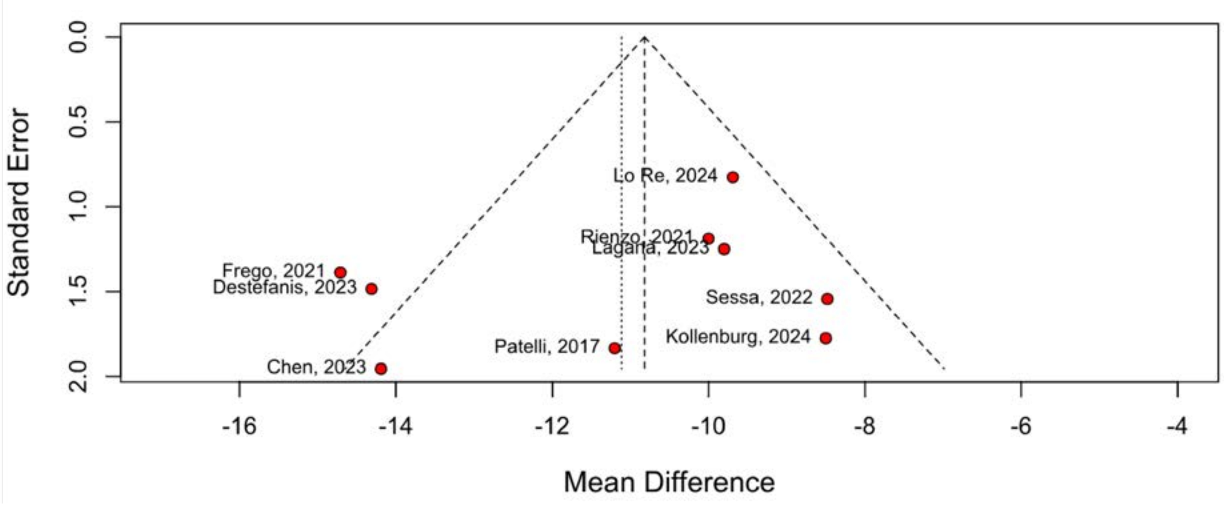

Abbreviations: TPLA – Transperineal Prostate Laser Ablation; IPSS – International Prostate Symptom Score; MD – Mean Difference; SE – Standard Error; CI – Confidence Interval.

Figure S10. Funnel plot for IPSS change at 6 months after TPLA. A broadly symmetric distribution is observed around the summary effect, without a clear directional pattern of small studies. The wider spread among less precise studies is expected and may reflect variability in power/energy settings and fibers-per-lobe across studies.

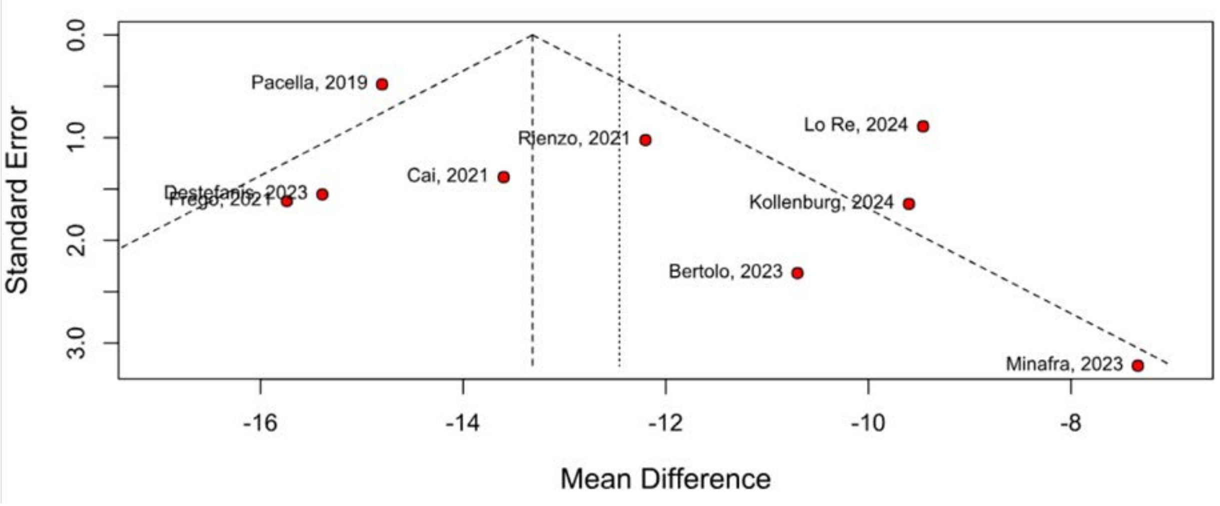

Abbreviations: TPLA – Transperineal Prostate Laser Ablation; IPSS – International Prostate Symptom Score; MD – Mean Difference; SE – Standard Error; CI – Confidence Interval.

Figure S11. Funnel plot for PVR change at 3 months after TPLA. No marked visual asymmetry is evident. The dispersion among smaller studies likely reflects clinical and technical heterogeneity (e.g., energy per fiber, inter-fiber spacing). Caution is warranted due to the limited number of contributing studies.

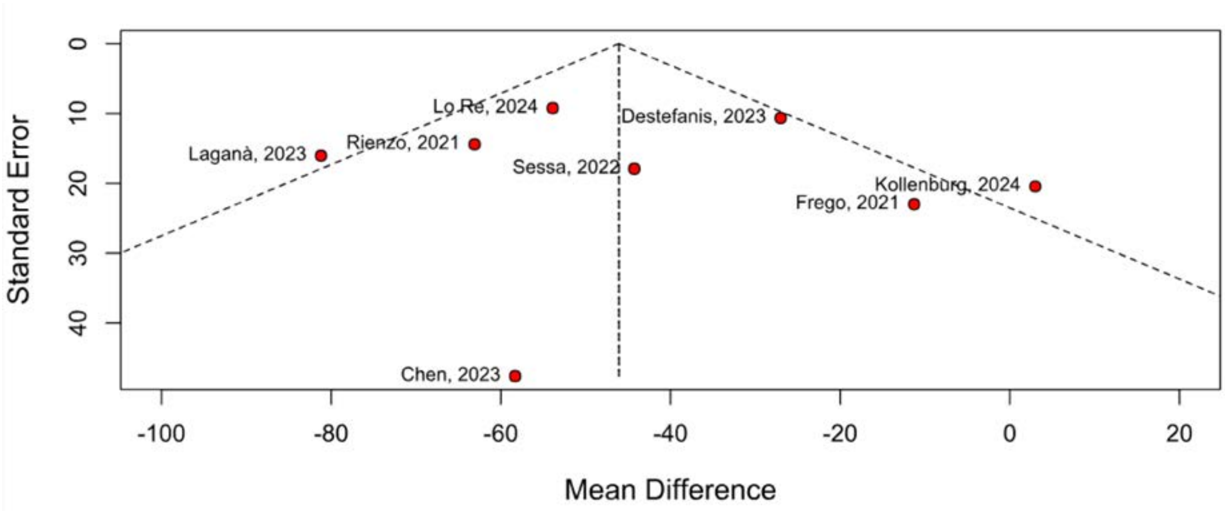

Abbreviations: PVR – Post-Void Residual; TPLA – Transperineal Prostate Laser Ablation; MD – Mean Difference; SE – Standard Error; CI – Confidence Interval.

Figure S12. Funnel plot for PVR change at 6 months after TPLA. The plot shows approximate symmetry around the pooled estimate with a typical funnel shape. Any subtle imbalance in the wings is insufficient to assert publication bias and may instead indicate heterogeneity of technique and perioperative protocols.

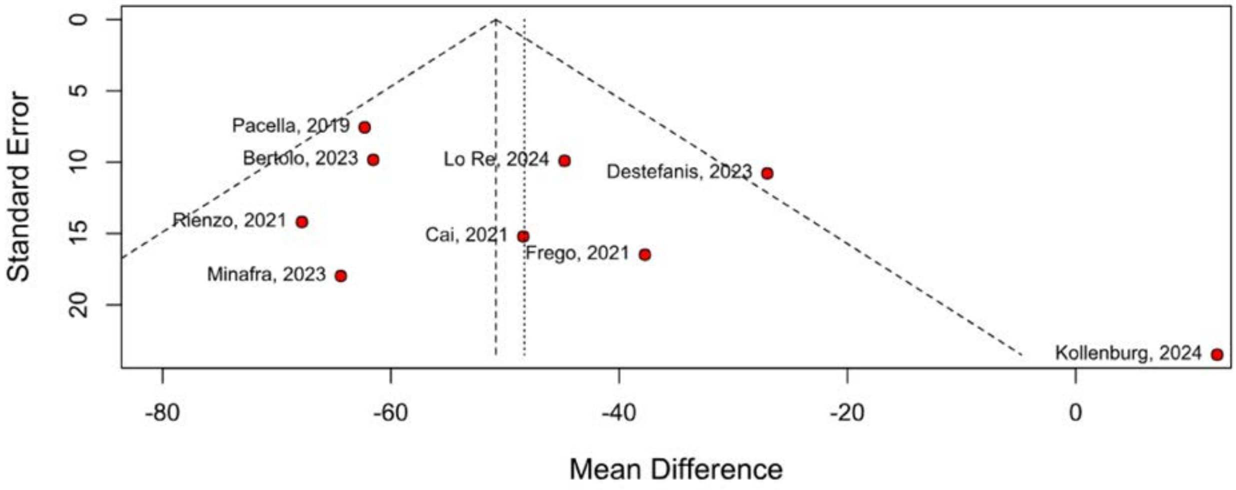

Abbreviations: PVR – Post-Void Residual; TPLA – Transperineal Prostate Laser Ablation; MD – Mean Difference; SE – Standard Error; CI – Confidence Interval.

Figure S13. Funnel plot for Qmax change at 6 months after TPLA. Visual inspection suggests near-symmetric scatter; however, a slightly broader spread among less precise studies is noted, consistent with methodological/technical variability. Small-study effects cannot be ruled out.

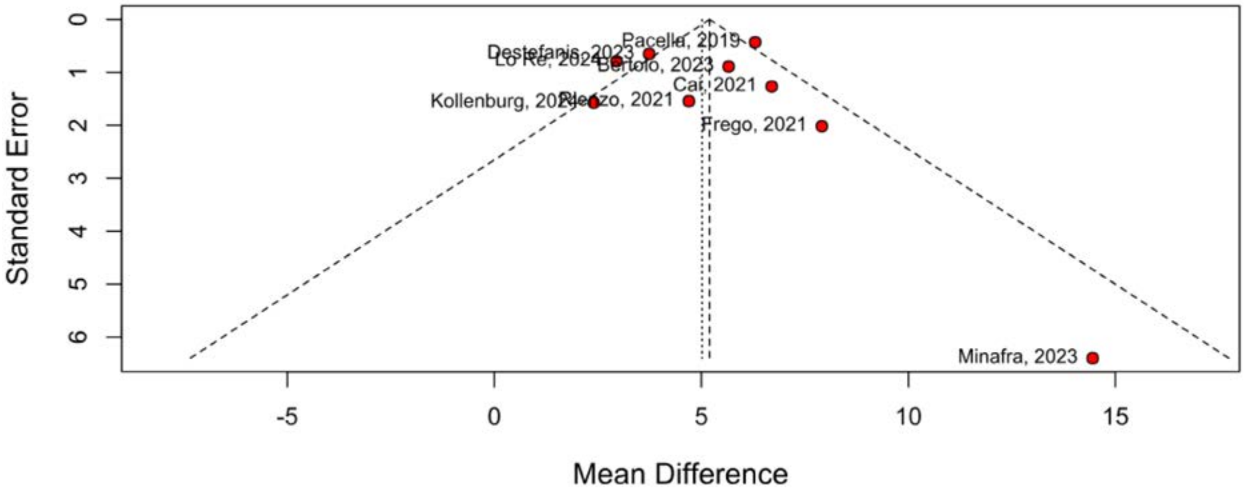

Abbreviations: Qmax – Maximum Urinary Flow Rate; TPLA – Transperineal Laser Ablation; MD – Mean Difference; SE – Standard Error; CI – Confidence Interval.

Figure S14. Funnel plot for Qmax changes 12 months after TPLA. The distribution is broadly symmetric with few studies near the base of the funnel, limiting the ability to detect asymmetry. Findings should be interpreted with caution given sample size and heterogeneity across techniques and follow-up schedules.

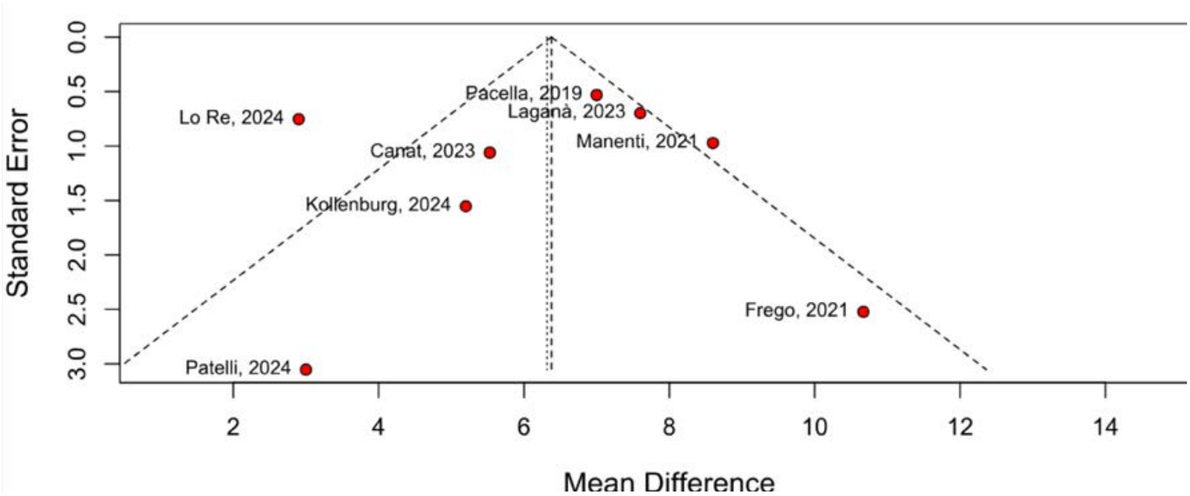

Abbreviations: Qmax – Maximum Urinary Flow Rate; TPLA – Transperineal Laser Ablation; MD – Mean Difference; SE – Standard Error; CI – Confidence Interval.

Table S1. PRISMA 2020 checklist of items for systematic reviews. Checklist of 27 items used to ensure transparency and completeness of reporting according to PRISMA 2020 guidelines.

Section and Topic	Item #	Checklist item	Location where item is reported
**TITLE**			
Title	1	Identify the report as a systematic review.	Page 01
**ABSTRACT**			
Abstract	2	See the PRISMA 2020 for Abstracts checklist.	Page 02
**INTRODUCTION**			
Rationale	3	Describe the rationale for the review in the context of existing knowledge.	Page 03
Objectives	4	Provide an explicit statement of the objective(s) or question(s) the review addresses.	Page 03
**METHODS**			
Eligibility criteria	5	Specify the inclusion and exclusion criteria for the review and how studies were grouped for the syntheses.	Page 04
Information sources	6	Specify all databases, registers, websites, organisations, reference lists and other sources searched or consulted to identify studies. Specify the date when each source was last searched or consulted.	Page 04
Search strategy	7	Present the full search strategies for all databases, registers and websites, including any filters and limits used.	Page 04
Selection process	8	Specify the methods used to decide whether a study met the inclusion criteria of the review, including how many reviewers screened each record and each report retrieved, whether they worked independently, and if applicable, details of automation tools used in the process.	Page 04
Data collection process	9	Specify the methods used to collect data from reports, including how many reviewers collected data from each report, whether they worked independently, any processes for obtaining or confirming data from study investigators, and if applicable, details of automation tools used in the process.	Page 05
Data items	10a	List and define all outcomes for which data were sought. Specify whether all results that were compatible with each outcome domain in each study were sought (e.g. for all measures, time points, analyses), and if not, the methods used to decide which results to collect.	Page 05
	10b	List and define all other variables for which data were sought (e.g. participant and intervention characteristics, funding sources). Describe any assumptions made about any missing or unclear information.	Page 05
Study risk of bias assessment	11	Specify the methods used to assess risk of bias in the included studies, including details of the tool(s) used, how many reviewers assessed each study and whether they worked independently, and if applicable, details of automation tools used in the process.	Page 09
Effect measures	12	Specify for each outcome the effect measure(s) (e.g. risk ratio, mean difference) used in the synthesis or presentation of results.	Page 05
Synthesis methods	13a	Describe the processes used to decide which studies were eligible for each synthesis (e.g. tabulating the study intervention characteristics and comparing against the planned groups for each synthesis (item #5)).	Page 05
	13b	Describe any methods required to prepare the data for presentation or synthesis, such as handling of missing summary statistics, or data conversions.	Page 05
	13c	Describe any methods used to tabulate or visually display results of individual studies and syntheses.	Page 05
	13d	Describe any methods used to synthesize results and provide a rationale for the choice(s). If meta-analysis was performed, describe the model(s), method(s) to identify the presence and extent of statistical heterogeneity, and software package(s) used.	Page 05
	13e	Describe any methods used to explore possible causes of heterogeneity among study results (e.g. subgroup analysis, meta-regression).	Page 05
	13f	Describe any sensitivity analyses conducted to assess robustness of the synthesized results.	Page 05

Section and Topic	Item #	Checklist item	Location where item is reported
Reporting bias assessment	14	Describe any methods used to assess risk of bias due to missing results in a synthesis (arising from reporting biases).	Page 09
Certainty assessment	15	Describe any methods used to assess certainty (or confidence) in the body of evidence for an outcome.	Page 09
**RESULTS**			
Study selection	16a	Describe the results of the search and selection process, from the number of records identified in the search to the number of studies included in the review, ideally using a flow diagram.	Page 06
	16b	Cite studies that might appear to meet the inclusion criteria, but which were excluded, and explain why they were excluded.	Page 06
Study characteristics	17	Cite each included study and present its characteristics.	Page 06
Risk of bias in studies	18	Present assessments of risk of bias for each included study.	Page 09
Results of individual studies	19	For all outcomes, present, for each study: (a) summary statistics for each group (where appropriate) and (b) an effect estimate and its precision (e.g. confidence/credible interval), ideally using structured tables or plots.	Page 07
Results of syntheses	20a	For each synthesis, briefly summarise the characteristics and risk of bias among contributing studies.	Page 06
	20b	Present results of all statistical syntheses conducted. If meta-analysis was done, present for each the summary estimate and its precision (e.g. confidence/credible interval) and measures of statistical heterogeneity. If comparing groups, describe the direction of the effect.	Page 06
	20c	Present results of all investigations of possible causes of heterogeneity among study results.	Page 06
	20d	Present results of all sensitivity analyses conducted to assess the robustness of the synthesized results.	Page 07
Reporting biases	21	Present assessments of risk of bias due to missing results (arising from reporting biases) for each synthesis assessed.	Page 09
Certainty of evidence	22	Present assessments of certainty (or confidence) in the body of evidence for each outcome assessed.	Page 09
**DISCUSSION**			
Discussion	23a	Provide a general interpretation of the results in the context of other evidence.	Page 10
	23b	Discuss any limitations of the evidence included in the review.	Page 11
	23c	Discuss any limitations of the review processes used.	Page 11
	23d	Discuss implications of the results for practice, policy, and future research.	Page 11
**OTHER INFORMATION**			
Registration and protocol	24a	Provide registration information for the review, including register name and registration number, or state that the review was not registered.	Page 01
	24b	Indicate where the review protocol can be accessed, or state that a protocol was not prepared.	Page 04
	24c	Describe and explain any amendments to information provided at registration or in the protocol.	Page 04
Support	25	Describe sources of financial or non-financial support for the review, and the role of the funders or sponsors in the review.	Page 12
Competing interests	26	Declare any competing interests of review authors.	Page 12
Availability of data, code and other materials	27	Report which of the following are publicly available and where they can be found: template data collection forms; data extracted from included studies; data used for all analyses; analytic code; any other materials used in the review.	Page 05

From: Page MJ, McKenzie JE, Bossuyt PM, Boutron I, Hoffmann TC, Mulrow CD, et al. The PRISMA 2020 statement: an updated guideline for reporting systematic reviews. BMJ 2021;372:n71. doi: 10.1136/bmj.n71. This work is licensed under CC BY 4.0. To view a copy of this license, visit https://creativecommons.org/licenses/by/4.0/

Abbreviations: PRISMA – Preferred Reporting Items for Systematic Reviews and Meta-Analyses.

Table S2 Technical parameters and perioperative data of the included studies. Overview of procedural characteristics, including anesthesia type, laser settings, number of fibers, and perioperative management strategies.

Study author/ year	Anesthesia	Laser system	Power setting (W)	Energy setting	Number and type of needles	Minimum distance from bladder neck (mm)	Minimum distance from the urethra (mm)	Minimum distance between Needles (mm)	No of fibres	Procedure time (min)	Ablation time (min)	Hospitalization	Catheterization time (days)	Antibiotic prophylaxis	Therapy at discharge	Pre-Op BPH Therapy N (%)	Antiaggregant/ Anticoagulant therapy N (%)
**Bertolo et al. (23), 2023**	Standard spinal anesthesia	SoracteLite EchoLaser, Elesta	4.5 reduced to 3.5 after 1-2 min	1800 J/ fiber/firing	1 or 2 needles per lobe 21 G	15	8	10	1 each needle	TPLA 35 (30-55) / TURP 68 (60-95)	NA	TPLA 2 (2-3) days / TURP 3 (3-4) days	TPLA 4 (2-7) / TURP 3 (3-4)	NA	NA	23 (88.5) / 22 (88)	10 (38.5%) / 0
**Cai et al. (24), 2021**	Local anesthesia (2% lidocaine)	SoracteLite EchoLaser, Elesta	3	1800 J/ fiber/firing	1 needle per lobe 21 G	15	8	15	1 each needle	60.9 (10.8)	42.6 (9.9)	1.5 (0.5) hours	16.5 (4.2)	NA	NA	NA	NA
**Canat et al. (25), 2023**	Sedation (Midazolam) + Local anesthesia (2% lidocaine)	SoracteLite EchoLaser, Elesta	NA	1800 J/ fiber/firing	1 needle per lobe 1 more needle if PV >60 mL 21 G	15	8	NA	2 (2-2.5)[1]	16 (2.35)	NA	TPLA 0.67 ±0.49 days / TURP 1.27 ±0.46 days	TPLA 6.5 (1.02) / TURP 2.27 (0.46)	NA	Dexamethasone and non-steroidal anti-inflammatory	NA	9 (36%) / 0
**Chen et al. (21), 2023**	Local anesthesia (2% lidocaine)	Asclepion Laser Technologies GmbH	3-5	1800 J/ fiber/firing	1 needle per lobe 21 G	20	8	10	1 each needle	TPLA 60.1 (19.67) / TURP 111 (49.09)	TPLA 36 (16) / TURP 100.63 (48.51)	TPLA 2.5 (0.52) days / TURP 2.94 (0.57) days	TPLA 11.38 (2.81) / TURP 8.88 (2.22)	NA	Antibiotic therapy for 7 days.	NA	NA
**Deste-fanis et al. (26), 2023**	Optional sedation + Local anesthesia (lidocaine/ bupivacaine)	SoracteLite EchoLaser, Elesta	NA	NA	Multiple 21 G Chiba needles	NA	NA	NA	2 (2-2.5)[1]	42.5 (35-50)	17.5 (8.5-18.5)	NA	NA	According to preop urine culture	NA	a-blockers 12 (30%) 5-ARIs 4 (10%) Combined therapy 15 (37.5%)	22 (55%)
**Frego et al. (27), 2021**	Sedation (Midazolam) + Local anesthesia (2% lidocaine)	SoracteLite EchoLaser, Elesta	3	1800 Joule/ fiber/firing in 600 s	1 needle per lobe 1 more needle if PV 60 mL 21 G Chiba	15	10	10	1 each needle	NA	17.21 (10-18.8)	1 day	7	Levofloxacin 500 mg (1 day before and for 5 days after procedure)	Antibiotic for 5 days; Dexamethasone 8 mg and Ketoprofen 100 mg for 7 days	a-blockers 22 (100%); Combined therapy 6 (27.3%)	NA
**Kollen-burg et al. (28), 2024**	Optional sedation (Midazolam) + Local anesthesia (2% lidocaine)	SoracteLite EchoLaser, Elesta	3	1800 J/ fiber/firing	1 needle per lobe	15	8	NA	1 each needle	59 (14)	17.2 (6.3)	6.5 (5.0) hours	15.2 (3.5)	Ciprofloxacin 500 mg single dose	Dexamethasone 8 mg for 7 days	a-blockers 12 (60%); 5-ARIs 8 (40%) B3-agonist 1 (5)	NA
**Laganà et al. (29), 2023**	Sedation + Local anesthesia	SoracteLite EchoLaser, Elesta	3	1800 J/ fiber/firing	1-2 needles per lobe 21 G	15	8	10	NA	48.8 (14.3)	13 (1.95)	NA	14.9 (7.5)	2 g cephazolin IV (before the start of the procedure)	NA	a-blockers 27 (42.9%) 5-ARIs 6 (9.5%)	NA
**Lo Re et al. (30), 2024**	Sedation (Oral BZD*) + Local anesthesia (2% lidocaine)	SoracteLite EchoLaser, Elesta	5 reduced to 3.5 after 2 min	NA	1-2 needles 21 G	15	8	NA	2 (2-2)[1]	NA	NA	99% of patients were discharged within daily hospital stay.	7 (7-7)	NA	NA	a-blockers 60 (60%) 5-ARIs 4 (4%); Combined therapy 17 (17%)	24 (24%)
**Manenti et al. (31), 2021**	Local anesthesia with 2% lidocaine	SoracteLite EchoLase, Elesta	5 reduced to 3 after 2 min	1800 J/ fiber/firing	1 needle per lobe 1 more needle if PV >45 mL 21 G Chiba	15	10	8	1 each needle	28.2 (10.6)	NA	NA	NA	Levofloxacin 500 mg (1 h before and for 5 days after the procedure).	Antibiotic for 5 days; Acetaminophen 1000 mg if necessary; Prednisone 25 mg for 5 days with subsequent dose tapering; Alpha-blockers for 30 days	Combined therapy 44 (100%)	NA

Study author/ year	Anesthesia	Laser system	Power setting (W)	Energy setting	Number and type of needles	Minimum distance from bladder neck (mm)	Minimum distance from the urethra (mm)	Minimum distance between Needles (mm)	No of fibres	Procedure time (min)	Ablation time (min)	Hospitalization	Catheterization time (days)	Antibiotic prophylaxis	Therapy at discharge	Pre-Op BPH Therapy N (%)	Antiaggregant/ Anticoagulant therapy N (%)
**Minafra et al. (32), 2023**	Sedation + Local anesthesia (2% lidocaine)	SoracteLite EchoLase, Elesta	NA	NA	1-2 needles per lobe	15	8	NA	1 each needle	NA	NA	NA	NA	NA	NA	NA	NA
**Pacella et al. (33), 2019**	Sedation (Midazolam) + Local anesthesia (2% lidocaine)	SoracteLite EchoLase, Elesta	3	1800 J/ fiber/firing	1 needle per lobe 1 more needle if PV > 40 mL 21 G Chiba	15	8	8	1 each needle	44.1 (12.9)	23.4 (10.2)	1.8 (0.4) days	11.3 (11.5)	Ciprofloxacin 500 mg single dose	NA	NA	NA
**Patelli et al. (34), 2017**	Sedation (Midazolam) + Local anesthesia (2% lidocaine)	SoracteLite EchoLase, Elesta	3	1200-1800 J/fiber/ firing	1-2 needles per lobe 21 G Chiba	15	8	15	1 each needle	43.3 (8.7)	15.9 (3.9)	1.5 (0.4) days	17.3 (10.0)	Antibiotic therapy from the previous day and for a variable period	NA	NA	NA
**Patelli et al. (35), 2024**	Sedation (Midazolam) + Local anesthesia (2% lidocaine)	SoracteLite EchoLase, Elesta	3	NA	1 needle per lobe 21 G	NA	NA	NA	3.5[2]	43.4 (8.7)	17.3 (4.4)	NA	22.8 (10.9)	Levofloxacin (initiated the day before and 7 days after procedure)	NA	16 (100%)	NA
**Polve-rino et al. (36), 2023**	Sedation (Oral BZD[a]) + Local anesthesia (2% lidocaine)	SoracteLite EchoLase, Elesta	NA	NA	NA	NA	NA	NA	2 (2-2)[1]	NA	NA	1	7 (7-9)	NA	NA	NA	18 (78%)
**Rienzo et al. (37), 2021**	Sedation + Local anesthesia (2% lidocaine)	SoracteLite EchoLaser, Elesta	4.5 reduced to 3.5 after 1–2 min	1800 J/ fiber/firing	1 needle per lobe 1 more needle if PV > 55–60 mL 21 G Chiba	15	8	10	2.2 (0.5)[2]	36 (9.5)	NA	20.8 (3.6) hours	8.7 (2.5)	Oral cephalo-sporines or fluoroqui-nolones (1 h before and for 7 days after procedure)	Antibiotic for 5 days; prednisone 25 mg for 15 days with subsequent tapering of the dose; bromelain for 30 days; Alpha-blockers for 30 days	a-blockers 14 (66.7%) 10 5-ARI (47.6%) 8 combination therapy (38.1%)	NA
**Sessa et al. (38), 2022**	Sedation (Oral BZD solution) + Local anesthesia (2% lidocaine and lidocaine-prilocaine 5% cream)	SoracteLite EchoLase, Elesta	5 reduced to 3.5 after 2min	1400 J/ fiber/ firing [b]	1 needle per lobe 21 G Chiba	15	8	NA	2 (2-2)[1]	31.5 (28-37)	NA	6.4 (5.9-7.2) hours	7 (7-7)	2 g cephazolin IV (1h before the start of the procedure)	Antibiotic (cefixime 400 mg daily), for 7 days; Gastroprotective therapy (pantoprazole 20 mg daily) for 7 days; Ibuprofen 600 mg twice a day for 7 days	a-blockers 20 (52.6%) 6 5-ARI (15.8%) combination therapy 5 (10.5%).	11 (28.9%)

Values are presented in absolute numbers unless otherwise specified. 5-ARI, 5-alpha reductase inhibitor; BZD, benzodiazepine; G, Gauge; IV, intravenous; NA, Non available; PV, Prostate volume; TPLA, Transperineal laser ablation; TURP, transurethral resection of the prostate; W, Watt.

[1]median, fiber per patient.

[2] mean, fiber per patient.

[a] According to patients' preference.

[b] In the case of large prostates, additional laser energy was delivered up to 1800J per fiber.

Table S3. Inclusion and exclusion criteria of the included studies. Detailed eligibility criteria applied in each study, including design, recruitment period, patient characteristics, and follow-up duration.

Study author/year	Design	Recruitment period	Inclusion criteria	Exclusion criteria	Follow-up	N (TPLA/TURP)
**Bertolo et al. (23), 2023**	RCT (TPLA versus TURP); Open-label; Monocentric	Jan 2020-Sep 2021	Age 18-75<br>Normal ejaculatory function and presence of antegrade ejaculation before surgery<br>IPSS ≥ 10<br>Qmax <15 mL/s<br>PV <100mL<br>Normal pre-operatory urine analysis	Previous prostate surgery, history of PCa or urethral stricture, Marion's disease, concomitant bladder stones, presence of median obstructive lobe, and neurological disorders*.	6 months	51 (26/25)
**Cai et al. (24), 2021**	Non comparative; Retrospective; Monocentric	June 2018-Jan 2020	Age >50<br>PV ≥ 30mL<br>PVR 50-400<br>IPSS ≥12<br>Qmax <=15	Previous prostate, bladder neck, or urethral surgery, PSA >4 ng/mL, diagnosed PCa, severe urethral stricture, neurological disorders*, hypersensitivity to ultrasound contrast media.	6 months	20
**Canat et al. (25), 2023**	RCT (TPLA versus TURP); Monocentric	Nov 2021-Feb 2023	Age ≥ 50<br>IPSS >12<br>Qmax ≤ 15 mL/s<br>TURP candidates	Urethral stricture, previous bladder or prostate surgery, bladder dysfunction, long-standing urethral catheters, PCa, neurological disorders*, and patients who had undergone rectal surgery.	15 months	50 (25/25)
**Chen et al. (22), 2023**	RCT (TPLA versus TURP); Open-label; Monocentric	Jun 2019-Dec 2021	Age >50<br>IPSS >8<br>PV 30-100mL<br>Qmax ≤ 15 mL/s<br>RUV ≥ 50 mL<br>Failure with prior treatment or patients who were unsuitable for medical treatment as judged by the clinician	Urethral stenosis, previous prostate, bladder or urethral surgery, bladder calculi or tumor, PCa, PSA >4 ng/ml, neurological disorders*, post-rectal surgery or patients with anal atresia, severe coagulation disorders or infection	12 months	51 (25/26)
**Destefanis et al. (26), 2023**	Non comparative; Prospective; Monocentric	Oct 2020-June 2022	High hemorrhagic risk due to ongoing pharmacological therapy or due to pre-existent diseases<br>ASA score > 3<br>Indwelling bladder catheter or intermittent catheterization<br>IPSS > 8<br>Qmax < 15 ml/s	Clinical suspicion of hypo or non-contractile bladders, PVR >500mL, PCa, urethral stricture, PV <30mL, previous prostate, bladder or bladder neck surgery, neurological disorders* or cognitive impairment	6 months	40
**Frego et al. (27), 2021**	Non comparative; Prospective; Monocentric	July 2019-Jan 2020	Age ≥ 45<br>IPPS ≥ 8<br>PSA < 4 ng/mL, previous negative prostate biopsy, or negative DRE<br>Qmax 15 mL/s<br>PVR ≤ 150 mL<br>PV 30–100 mL	Previous bladder neck, urethral or prostatic surgery, previous diagnosis of BCa or PCa, neurological disorders*, gross hematuria, active UTI	12 months	22
**Kollenburg et al. (28), 2024**	Non comparative; Prospective; Multicentric	NA	Age ≥ 45<br>IPSS ≥8<br>PSA < 4 ng/mL or PSA >4 ng/ml with previously negative prostate biopsy and negative DRE<br>Qmax ≤ 15ml/s<br>PVR ≤ 150mL<br>PV 30-100mL	Prostate, bladder neck or urethral surgery, PCa or BCa, neurological disorders*, acute UTI or macroscopic hematuria	12 months	20
**Lagana et al. (29), 2023**	Non comparative; Prospective; Monocentric	Jan 2020-Jan 2022	Desire to spare antegrade ejaculation<br>Intolerance of poorly compliant to medical therapy, with no indication for surgery.	Acute and chronic prostatitis, prior prostatic abscess, PV>85 mL, PSA>4.0 ng/mL without a negative MRI scan or negative biopsy for PCa	12 months	63
**Lo Re et al. (30), 2024**	Non comparative; Prospective; Monocentric	April 2021-July 2023	Age ≥ 45<br>IPPS ≥ 8<br>PV 30-100 mL<br>Lack of efficacy, intolerance, or poor compliance to previous medical therapy or strong desire to preserve antegrade ejaculation or very high risk for standard surgery due to comorbidities	Clinical suspicion or previous PCa, neurologic disorders*, urethral strictures, bladder stones, large median lobe, previous prostatic surgery	Baseline, 3, 6, 12 months, last follow up	100
**Manenti et al. (31), 2021**	Non comparative; Prospective; Monocentric	May 2018-Feb 2020	Age > 50<br>IPPS ≥12<br>PV > 30 mL<br>lack of efficacy, intolerance, or poor compliance to previous medical therapy	Urethral stricture, previous prostatic surgery, clinical or imaging findings suspicious for malignancy confirmed by biopsy, neurological disorders*, large median lobe, indwelling catheter, previous diagnosis of BCa or PCa	12 months	44

Study author/year	Design	Recruitment period	Inclusion criteria	Exclusion criteria	Follow-up	N (TPLA/TURP)
**Minafra et al. (32), 2023**	Non comparative; Retrospective; Monocentric	Sep 2018-Mar 2019	IPPS ≥ 12<br>Qmax ≤ 15 mL/s<br>PV 30-100 mL (TRUS)	Previous treatment for BPO, history of bladder neck or urethral surgery, indwelling catheter or intermittent catheterization, bladder stones, detrusor acontractility or severely impaired contractility, urethral strictures, neurological disorders*, active UTI, macroscopic hematuria, history of clinical suspicion of PCa (elevated PSA without a negative prostate biopsy or negative DRE), history or clinical suspect of BCa.	3 years	21
**Pacella et al. (33), 2019**	Non comparative; Retrospective; Multicentric	NA	Age > 50<br>IPSS ≥ 12<br>PV > 30 mL (TRUS)<br>Qmax < 15 mL/s<br>PVR < 400 ml	Urethral stricture, previous prostatic surgery, neurological disorders*, previous diagnosis of PCa	12 months	160
**Patelli et al. (34), 2017**	Non comparative; Prospective; Monocentric	May 2014-May 2016	Age > 50<br>IPSS ≥ 13<br>PV > 30 mL (TRUS)<br>Qmax ≥ 5 to ≤ 15 mL/s<br>PVR ≥ 50 ml	Urethral stricture, previous prostate, bladder neck, or urethral surgery, PCa or PSA >4 ng/mL. neurological disorders*.	3 months	18
**Patelli et al. (35), 2024**	Non comparative; Prospective; Monocentric	May 2014-Sep 2018	Age > 50<br>IPSS ≥ 13<br>PV > 30 mL (TRUS)<br>Qmax ≥ 5 to ≤ 5 mL/s<br>PVR > 50 ml	Urethral stricture; PCa or suspected neoplastic disease; known neurological disorders*	36 months and last follow up	40
**Polverino et al. (36), 2023**	Non comparative; Prospective; Monocentric	April 2021-Feb 2023	ASA score ≥ 3<br>IPSS ≥ 8<br>PV 30-100 mL	NA	12 months	23
**Rienzo et al. (37), 2021**	Non comparative; Prospective; Monocentric	Sep 2018-Mar 2019	Age 40–90<br>IPSS ≥ 12<br>PV ≥ 100 mL<br>lack of efficacy, intolerance, or poor compliance to previous medical therapy	Previous surgical treatment for BPH, indwelling catheter or intermittent catheterization, bladder stones, detrusor acontractility or hypocontractility (BCI < 50), urethral strictures, neurological disorders*, previous diagnosis of BCa of PCa	6 months	21
**Sessa et al. (38), 2022**	Non comparative; Prospective; Monocentric	April 2021-Feb 2022	Age ≥ 45<br>IPPS ≥ 12<br>PV 30-100 mL (TRUS)<br>lack of efficacy,bintolerance, or poor compliance to previous medical therapy	Clinical suspicion or previous PCa, neurological disorders*, urethral strictures, bladder stones, indwelling catheter with severe detrusor hypo-contractility	1, 3 months and last follow up (4-12 months)	38

Values are presented in absolute numbers unless otherwise specified. ASA, American Society of Anesthesiologists; BCa, Bladder cancer; BCI, Bladder contractility index; BPH, Benign prostatic obstruction; BPO, Benign prostatic obstruction; Dec, December; DRE, Digital rectal examination; Feb: February; IPPS, International Prostatic Symptoms Score; Jan: January; Mar: March; Mo, Months; MRI, Magnetic resonance imaging; NA, Non available; Nov, November; Oct, October; PCa, Prostate cancer; PSA, Prostate-specific antigen; P, Prostate volume; PVR, Post-Void Residual; Qmax, Maximum urinary flow; RCT, Randomized controlled trials; RUV, Residual urine volume; Sep, September; TPLA, Transperineal laser ablation; TRUS, Transrectal ultrasonographic images; TURP, transurethral resection of the prostate; UTI, urinary tract infection.*neurological disorders: e.g., multiple sclerosis, Parkinson's disease, or known history of spinal cord injury.

Table S4. Reported postoperative complications and management strategies among included studies. Complication rates are stratified by Clavien-Dindo classification. Most complications were mild (Grade I-II) and included transient urinary retention, infection, and hematuria.

Study author/year	N	Type and number of complications	Management	Complication rate: N (%)	Clavien-Dindo: N (%)
**Bertolo et al. (23), 2023**	51	No complications	NA	0 (0)	NA
**Cai et al. (24), 2021**	20	Intraoperative urethral burn: 1<br>Transient urinary retention: 1	Bladder catheter for 25 days	2 (10)	NA
**Canat et al. (25), 2023**	50	TPLA<br>No complications<br>TURP<br>Urethral stenosis: 1	NAP	NA	NA
**Chen et al. (22), 2023**	51	TPLA<br>Urinary retention: 1<br>Prostate abscess: 1<br>Overactive bladder: 1<br>Urinary infection: 1<br>TURP<br>Electrolyte disturbance: 1<br>Urge-incontinence: 1<br>Hematuria: 1<br>Urinary infection: 1	Oral medications and supportive treatment	TPLA<br>4 (16)<br>TURP<br>5 (19.23)	NA
**Destefanis et al. (26), 2023**	40 (3 months) / 38 (6 months)	Catheter displacement or malfunction: 3<br>Urinary tract infection: 5<br>Hematuria: 1<br>Acute urinary retention: 13<br>Blood transfusion: 1<br>Acute heart failure: 3.		19 (47,5)	Grade I: 16 (40)<br>Grade II: 9 (22.5)<br>Grade III: 1 (2.5)
**Frego et al. (27), 2021**	22	Dysuria: 8<br>Acute urinary retention: 3<br>Urinary infection: 2	Antibiotics therapy	13 (59)	Grade I: 8 (36.3)<br>Grade II: 5 (22.7)
**Kollenburg et al. (28), 2024**	20	Dysuria: 5<br>Urgency: 4<br>Haematuria: 3<br>Pain: 2<br>Frequency: 1<br>Urinary retention: 10<br>Urinary tract infection: 7	Conservative treatment and antibiotics		Grade I:<br>Dysuria: 5 (25)<br>Urgency: 4 (20)<br>Haematuria: 3 (15)<br>Pain: 2 (10)<br>Frequency: 1 (5)<br><br>Grade II:<br>Urinary retention: 10 (50)<br>Urinary tract infection: 7 (35)
**Laganà et al. (29), 2023**	63	Prostatic abscess: 2<br><br>Orchitis: 1	Abscess drainage<br><br>Antibiotics	3 (4,8)	Grade II: 1 (1,6)<br><br>Grade IIIa: 2 (3.2)
**Lo Re et al. (30), 2024**	100	Urinary infection: 2	Oral antibiotics	2 (2)	Grade II: 2 (2)
**Manenti et al. (31), 2021**	44	Urinary blockage and urinary clots: 5	Catheter reposition	5 (11,3)	NAP
**Minafra et al. (32), 2023**	21	No patient complained about the development of late-onset complications	NAP	NAP	NAP
**Pacella et al. (33), 2019**	160	Transient hematuria: 3<br>Acute urinary retention: 3<br>Orchitis: 1<br>Prostatic abscess: 1	None<br>Bladder catheter for 15 days<br>Antibiotic<br>Antibiotic and percutaneous drainage	8 (5)	Grade I: 7 (4.3)<br>Grade III: 1 (0.6)

Study author/year	N	Type and number of complications	Management	Complication rate: N (%)	Clavien-Dindo: N (%)
**Patelli et al. (34), 2017**	18	0	NAP	NAP	NAP
**Patelli et al. (35), 2024**	40	Prostatitis: 1<br>UTI: 1	NA	NA	NA
**Polverino et al. (36), 2023**	23	NA	NA	NA	Grade ≥2: 0 (0)
**Rienzo et al. (37), 2021**	21	Prostatic abscess: 1	Transperineal drainage and antibiotic	1 (4.7)	Grade III: 1 (4.7)
**Sessa et al. (38), 2022**	38	Acute urinary retention: 2	NA	NA	NA

NAP: not applicable; NA: not available

Table S5. GRADE assessment of the certainty of evidence across outcomes. Certainty of evidence graded according to GRADE domains (risk of bias, inconsistency, indirectness, imprecision, and publication bias). All main outcomes demonstrated high certainty.

Author(s): Iago Zang Pires, Marília Oberto da Silva Gobbo, Tanize Louize Milbradt, Renan Yuji Ura Sudo, Mable Pereira, Nilson Marquardt Filho, Márcio Augusto Averbeck.
Question: Transperineal Prostate Laser Ablation compared to Transurethral Resection of the Prostate for Benign Prostatic Enlargement

Certainty assessment							№ of patients		Effect		Certainty	Importance
№ of studies	Study design	Risk of bias	Inconsistency	Indirectness	Imprecision	Other considerations	Transperineal Prostate Laser Ablation	Transurethral Resection of the Prostate	Relative (95% CI)	Absolute (95% CI)		
**International Prostate Symptom Score changes (assessed with: points; Scale from: 0 to 35)**												
3	Randomised trials	Not seri-ous	Not serious	Not serious	Not serious	None	76	76	-	Mean 1.81 Pontos more (2.14 fewer to 5.76 more)	⊕⊕⊕⊕ High	CRITICAL
**Maximum urinary flow rate changes (assessed with: ml/s)**												
3	Randomised trials	Not serious	Not serious	Not serious	Not serious	None	76	76	-	Mean 10.73 ml/s lower (17.55 lower to 3.92 lower)	⊕⊕⊕⊕ High	CRITICAL
**Male Sexual Health Questionnaire for Ejaculatory Dysfunction changes (assessed with: points)**												
3	Randomised trials	Not serious	Not serious	Not serious	Not serious	None	76	76	-	Mean 4.78 pontos higher (0.65 higher to 8.91 higher)	⊕⊕⊕⊕ High	CRITICAL
**Simplified International Index of Erectile Function changes (assessed with: points)**												
3	Randomised trials	Not seri-ous	Not serious	Not serious	Not serious	None	76	76	-	Mean 0.17 pontos lower (1.89 lower to 1.55 higher)	⊕⊕⊕⊕ High	CRITICAL

CI: confidence interval

Abbreviations: GRADE – Grading of Recommendations, Assessment, Development and Evaluation.
